# Sanfilippo syndrome: consensus guidelines for clinical care

**DOI:** 10.1186/s13023-022-02484-6

**Published:** 2022-10-27

**Authors:** Nicole Muschol, Roberto Giugliani, Simon A. Jones, Joseph Muenzer, Nicholas J. C. Smith, Chester B. Whitley, Megan Donnell, Elise Drake, Kristina Elvidge, Lisa Melton, Cara O’Neill, MPS III Guideline Development Group

**Affiliations:** 1 Department of Pediatrics, International Center for Lysosomal Disorders (ICLD), University Medical Center Hamburg-Eppendorf, Hamburg, Germany; 2 DASA, Federal University of Rio Grande do Sul (UFRGS), Hospital de Clinicas de Porto Alegre (HCPA), Casa dos Raros, Porto Alegre, Brazil; 3 University of Manchester, Manchester, UK; 4 University of North Carolina at Chapel Hill, Chapel Hill, NC USA; 5 Department of Neurology and Clinical Neurophysiology, Women’s and Children’s Health Network and the Discipline of Paediatrics, University of Adelaide, Adelaide, Australia; 6 University of Minnesota, Minneapolis, MN USA; 7 Sanfilippo Children’s Foundation, Freshwater, NSW Australia; 8 Cure Sanfilippo Foundation, Columbia, SC USA

**Keywords:** Mucopolysaccharidosis type III, Sanfilippo syndrome, Diagnosis, Management, Recommendations

## Abstract

Sanfilippo syndrome is a group of rare, complex, and progressive neurodegenerative lysosomal storage disorders that is characterized by childhood dementia. The clinical management of patients with progressive neurological decline and multisystem involvement requires a multidisciplinary team with experience in the management of neurodegenerative disorders. Best practice guidelines for the clinical management of patients with these types of rare disorders are critical to ensure prompt diagnosis and initiation of appropriate care. However, there are no published standard global clinical care guidelines for patients with Sanfilippo syndrome. To address this, a literature review was conducted to evaluate the current evidence base and to identify evidence gaps. The findings were reviewed by an international steering committee composed of clinical experts with extensive experience in managing patients with Sanfilippo syndrome. The goal was to create a consensus set of basic clinical guidelines that will be accessible to and informed by clinicians globally, as well as providing a practical resource for families to share with their local care team who may not have experience with this rare disease. This review distills 178 guideline statements into an easily digestible document that provides evidence-based, expert-led recommendations for how to approach common management challenges and appropriate monitoring schedules in the care of patients with Sanfilippo syndrome.

**Supplementary Information:**

The online version contains supplementary material available at 10.1186/s13023-022-02484-6.

## Background

Sanfilippo syndrome (mucopolysaccharidosis type III [MPS III]) is a group of inherited lysosomal storage disorders, manifesting progressive central nervous system (CNS) and systemic disease in childhood, with progressive neurocognitive deterioration and loss of functional abilities, and premature death [1]. There are four autosomal recessive subtypes (types A, B, C, and D) of Sanfilippo syndrome. Each subtype is caused by a deficiency of a different enzyme that degrades the ubiquitous glycosaminoglycan (GAG) heparan sulfate (Table 1), which leads to substrate accumulation and cellular dysfunction [2].

**Table 1:** Classification and underlying enzyme deficiencies of Sanfilippo syndrome subtypes [39–42]

Disease subtype	Affected gene	Deficient enzyme	OMIM number
MPS IIIA	*SGSH*	Heparan-*N*-sulfatase	252900
MPS IIIB	*NAGLU*	*N*-acetyl-α-glucosaminidase	252920
MPS IIIC	*HGSNAT*	α-glucosaminidase *N*-acetyltransferase	252930
MPS IIID	*GNS*	*N*-acetylglucosamine 6-sulfatase	252940

*MPS* mucopolysaccharidosis, *OMIM* online Mendelian inheritance in man

The combined estimated prevalence of Sanfilippo syndrome (types A, B, C, and D) is between 1:50,000 and 1:250,000 depending on the population studied [3]. Sanfilippo syndrome type A is the most common subtype globally; however, the prevalence of subtypes can vary depending on region, with Sanfilippo syndrome type A being more prevalent in Northern Europe and Eastern Europe than in Mediterranean countries [4–6]. In contrast, Sanfilippo syndrome type B is the most prevalent subtype in Southern Europe [4, 7]. Sanfilippo syndrome types C and D are much less common overall, with estimated global incidences of 1:1,500,000 and 1:1,000,000, respectively [1]. However, the total number of patients with Sanfilippo syndrome is most likely underestimated owing to delayed or missed diagnoses, particularly for the most slowly progressing phenotypes.

The age at onset and extent and rate of progression in patients with Sanfilippo syndrome vary greatly between those with different subtypes (ie types A, B, C, and D) and within those with the same subtype (eg type A only). The behavioral, cognitive, and physical findings in patients with Sanfilippo syndrome present as a clinical spectrum from early-onset, rapidly progressive disease with death in late childhood and adolescence, to slower progressing forms that present in later childhood with survival into adulthood. In rare cases, more indolent disease with onset in adulthood is also observed in patients with Sanfilippo syndrome [2]. The natural history of Sanfilippo syndrome, while traditionally considered across three broad symptomatic phases, remains variable between individuals and is best considered as a phenotypic continuum. Typical disease manifests in patients between 1 and 4 years of age with presentation of mild global developmental or speech delay, usually after a period of normal development with somatic manifestations such as recurrent ear, nose, and throat (ENT) disease and/or bowel disturbance [2]. Behavioral difficulties include: hyperactivity, (hyper) orality and/or preservative chewing, temper tantrums, lack of fear (for danger), disobedience or unresponsiveness to discipline, and destructive behavior [8–11]. Physical manifestations in patients with Sanfilippo syndrome can include musculoskeletal, respiratory, gastrointestinal, cardiovascular complications, vision and hearing loss, and dental issues; these manifestations can further exacerbate the neurocognitive and behavioral challenges in these patients [2, 9, 12]. In the later phase of Sanfilippo syndrome, patients show a decline in engagement with their environment, dementia, and progressive loss of motor function. Patients may develop seizures, dysphagia, and become fully bedridden [2, 13–15]. For patients with severe forms of Sanfilippo syndrome, death usually occurs within their second decade of life [2, 16–18]. In contrast, patients with attenuated phenotypes of the disorder have a more variable life span, in rare cases surviving into their seventh decade of life [14, 19].

Patient care requires a collaborative specialist health and community-based multidisciplinary team with experience in the management of Sanfilippo syndrome. There is currently no disease-modifying therapy available for patients with Sanfilippo syndrome. However, disease-specific therapies for Sanfilippo syndrome are being studied (including forms of enzyme replacement therapy, substrate reduction therapy, hematopoietic stem cell transplantation, and gene therapy), with some reaching the mid-to-late stages of clinical development. In lieu of these emergent therapies, management focuses on supportive interventions to maintain function, optimize ability, and maximize quality of life for patients with Sanfilippo syndrome and their families.

Best practice guidelines for the clinical management of rare diseases are critical to ensure prompt diagnosis and initiation of appropriate care. Such guidelines allow physicians and other healthcare professionals to make recommendations based on the best available evidence, to improve the consistency of diagnosis and clinical management across treatment centers, and to enable affected families to make informed decisions regarding therapy. For patients with Sanfilippo syndrome, there are currently no published, standard global clinical care guidelines.

Here, a collaboration between Cure Sanfilippo Foundation (USA) and Sanfilippo Children’s Foundation (Australia) was initiated in mid-2017 to investigate current best practice in the clinical management of patients with Sanfilippo syndrome. A literature review and gap analysis were conducted to evaluate the current evidence base, and the findings were reviewed by an international steering committee consisting of clinical experts with extensive experience in managing patients with Sanfilippo syndrome. The goal was to create a consensus set of basic clinical guidelines for patients with Sanfilippo syndrome that will be accessible to and informed by clinicians globally, as well as providing a practical resource for families to share with their local care team who may not have experience with this rare disease. Here, 178 guideline statements are distilled into an easily digestible document that provides recommendations based on evidence and consensus clinical expertise for how to approach common management challenges in the care of patients with Sanfilippo syndrome. This review is a first step in establishing basic care guidance and will require updates as Sanfilippo syndrome becomes further characterized and should new therapies become available.

## Methods

**Fig. 1:**
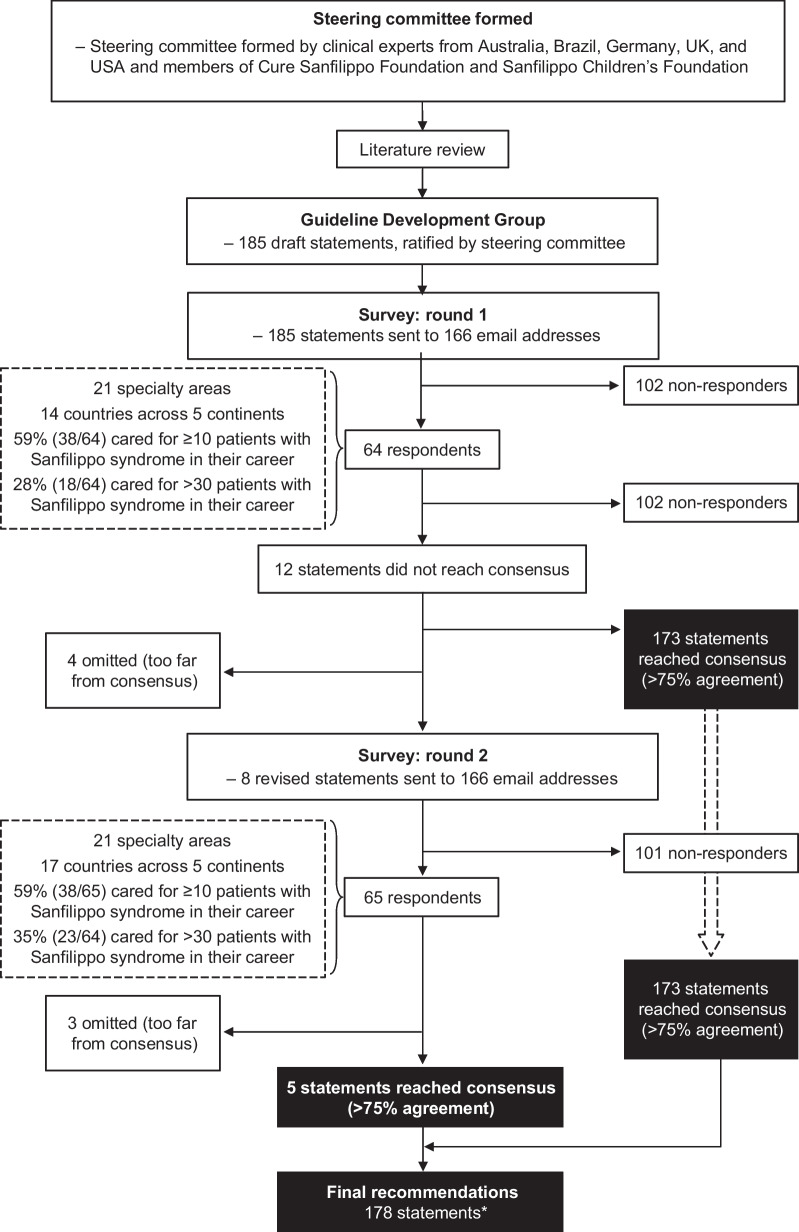
Flow chart for the development of consensus guideline statements. *One statement was subsequently refined by the steering committee during development of the guidelines

A consultative survey-based technique was used to reach consensus on best practice for the management of patients with Sanfilippo syndrome. An overview of the consensus process is shown in Fig. 1.

A steering committee was formed, consisting of clinical experts from Australia (including members of Sanfilippo Children’s Foundation), Brazil, Germany, the UK, and the USA (including members of Cure Sanfilippo Foundation), each with extensive experience in managing patients with Sanfilippo syndrome. A comprehensive literature review and gap analysis was conducted by members of the steering committee to consolidate the best available published information on the management of patients with Sanfilippo syndrome and to identify evidence gaps. The search terms used are detailed in the supplemental material. Publications reviewed in detail included any published article containing information on patients with Sanfilippo syndrome (types A, B, C, and D), including articles referencing mucopolysaccharidoses in general to delineate information specific to Sanfilippo syndrome.

A network of expert clinicians was invited to join a Guideline Development Group to consider the findings of the literature review and draft initial guideline statements for their area of expertise. In addition to the steering committee members, the Guideline Development Group included 29 clinicians (35 in total) with experience of Sanfilippo syndrome from nine countries (Additional file 1: Table S1). Collectively, the expert clinicians represented the following focus areas: neurology, metabolic and/or genetic diseases, orthopedics, gastroenterology, ophthalmology, cardiology, dentistry, ENT (including audiology), rehabilitative therapies (speech therapy, occupational therapy, behavioral therapy, and physical therapy), developmental pediatrics, anesthesiology, endocrinology, and integrative medicine (including nutrition and supplements). A total of 185 draft guideline statements were developed by the Guideline Development Group and refined or expanded upon by the steering committee. The draft guideline statements were sent to 166 clinicians from five continents with a survey asking them to rate their level of agreement with each statement on a 5-point Likert scale, as follows: Strongly agree, Agree, Neutral, Disagree, or Strongly disagree. They were also given the option ‘Not my area of expertise’ and asked to provide comments, particularly if they disagreed with a statement.

Consensus was defined as ≥ 75% of responses being ‘Strongly Agree’ or ‘Agree’, excluding responses of ‘Not my area of expertise’. This consensus threshold was determined by a literature review and applied to the rare disease field by the steering committee. The most common definition of consensus for Delphi studies is percent agreement, with ≥ 75% being the median threshold to define consensus [20]. No participants were compensated for their involvement.

## Results

Responses were received from 64 clinicians representing 21 specialty areas. Clinicians were based in 14 countries across five continents, as follows: 29.7% (n = 19) in North America, 26.6% (n = 17) in Europe, 23.4% (n = 15) in Australasia, 12.5% (n = 8) in South America, and 7.8% (n = 5) in Asia. Of the clinicians surveyed, 59% (n = 38) had cared for ≥ 10 patients with Sanfilippo syndrome in their career, and 28% (n = 18) had cared for > 30 patients with Sanfilippo syndrome. Consensus (defined as ≥ 75% responses of ‘Strongly Agree’ or ‘Agree’, excluding ‘Not my area of expertise’) was reached for 173 (94%) of 185 statements. After a steering committee review of the 12 non-consensus items, consensus on four of the statements was not reached and they were omitted. The remaining eight statements were revised based on comments from participants and recommendations from the steering committee. The eight revised were then distributed to the same global clinical email list. Of these eight statements, consensus was reached for five, while three were removed. A full list of all guideline statements and their level of consensus can be found in Additional file 1: Table S2.

Following the consensus-forming process, the steering committee convened to review the 178 guideline statements and discuss how to distill them into a practical and user-friendly format. As part of this process, consensus recommendations were divided into 156 core statements that tackle the most pressing needs faced by patients with Sanfilippo syndrome, and 22 supplemental statements that address some of the less common aspects of diagnosing and managing the disease, or areas that require further evidence. Additional refinement of the resulting guidance by the steering committee’s clinical experts was made in select areas, based on their collective clinical experience and consideration of any risks associated with recommended procedures. These few instances are noted where they occur.

### Optimal management relies on early diagnosis

Early diagnosis of Sanfilippo syndrome is critical to ensure the optimal care for patients and their families by enabling access to specific supportive interventions to maximize peak abilities, slow rate of decline, and improve quality of life. In addition to accessing appropriate education and developmental therapies, early diagnosis enables patients to participate in clinical trials and/or receive treatments as they emerge, and affords timely genetic counseling of affected families. However, diagnoses delayed by > 2 years are not uncommon in patients with Sanfilippo syndrome [21–24]. Potential reasons for delays include a lack of disease awareness, the absence or subtle presentation of somatic symptoms, and neurological symptoms that can be mistakenly considered as idiopathic developmental delays and behavioral challenges [23]. Furthermore, Sanfilippo syndrome is not included in newborn screening programs and patients often receive diagnoses such as idiopathic developmental delay, attention deficit hyperactivity disorder (ADHD), and/or autism without sufficient medical workup to identify Sanfilippo syndrome as the underlying genetic disorder [18, 25, 26].

A recent consensus-forming process identified eight signs and symptoms that presented early in neonates and infants that may, alone or in combination, raise suspicion of Sanfilippo syndrome [27]. Such signs and symptoms included coarse facial features, persistent hirsutism and/or prominent eyebrows, which have been reported as signs that are suggestive of Sanfilippo syndrome and should prompt referral to a metabolic physician and/or developmental specialist [27]. Somatic signs should also be viewed in the context of neurocognitive features. For example, early somatic signs that are not specific to Sanfilippo syndrome (eg frontal bossing and macrocephaly) become noteworthy when present alongside neurocognitive features (eg speech delay). Similarly, while episodic irritability and gastrointestinal discomfort, umbilical or inguinal hernia, and upper respiratory congestion are considered prevalent among neonates and infants [28], any of these conditions, including those listed previously, should raise suspicion of MPS diseases but particularly for Sanfilippo syndrome when present alongside the characteristic neurobehavioral features. Table 2 shows a list of neurological and somatic features that (alone or in combination) should raise suspicion of Sanfilippo syndrome. Sanfilippo syndrome should be considered in patients of all ages, not only young children, since slower progressing forms of the disorder are noted. For example, investigation for Sanfilippo syndrome is warranted in adults who show signs of early-onset dementia, vision loss with retinitis pigmentosa, and/or adult-onset cardiomyopathy [29]. Where such suspicion exists, screening and/or diagnostic tests should be initiated by the primary care provider to avoid delay of diagnosis, in conjunction with referral to an appropriate specialist.

**Table 2:** Clinical signs that should raise suspicion of Sanfilippo syndrome when present alone or in combination [9, 12, 27]

Type of clinical sign	Manifestation
*Neurological*	
Cognitive	Speech delay Non-specific developmental delay Intellectual disability with progressive loss of cognitive and daily living skills
Behavioral	Aggressive and/or destructive behavior Hyperactivity Hyperorality Obstinacy or temper tantrums Lack of fear (of danger) Disobedience/unresponsiveness to discipline Attention deficit hyperactivity disorder Motor restlessness Sensitivity to touch or temperature changes Autistic behaviors Sleep disturbance
Motor	Motor delays Gait disorders Spasticity
Other	Seizures
*Somatic*	
Craniofacial and physical appearance	Coarse facial features Coarse and thick hair Hirsutism Thickened skin Frontal bossing Macrocephaly
Abdominal/gastrointestinal	Colic-like episodes Diarrhea or chronic loose stools Constipation Gastrointestinal discomfort Umbilical or inguinal hernia Hepatosplenomegaly
Ear, nose, and throat	Hearing loss Recurrent otitis Requiring more than one set of ear grommets for persistent middle ear effusion or infection Chronic nasal congestion Need for earlier than usual adenotonsillectomy
Eyes	Retinitis pigmentosa
Heart	Arrhythmia Cardiomyopathy Valvular heart disease
Musculoskeletal	In-toeing Toe-walking Joint stiffness Osteonecrosis of the femoral head Scoliosis
Respiratory	Persistent tachypnea in the neonate Pneumonia Sleep apnea

### Confirming a diagnosis of Sanfilippo syndrome

In individuals with clinical features suggestive of Sanfilippo syndrome, confirmation of a diagnosis requires at least two biochemical or genetic markers of the disorder to be present: evidence of GAG accumulation (eg increased total GAGs or the more specific component substrate, heparan sulfate, in the urine or blood), decreased lysosomal enzyme activity, and/or evidence of pathogenic or likely pathogenic variants via molecular testing [30, 31].

#### GAG analysis

Analysis of urine GAGs using quantitative and qualitative approaches in a first morning void sample are accepted biochemical diagnostic tests, with a sample from a random time also being acceptable. A sterile sample is not required. Quantitative analysis of urine for the presence of GAG biomarkers using the spectrophotometric compound dimethylmethylene blue is often used as a first-line screen for MPS disorders [32–35]. The use of age-related reference ranges is strongly recommended owing to the natural decrease in GAG levels with age, both in affected patients and healthy individuals. The recommended qualitative GAG assay is GAG electrophoresis [31, 36, 37]. Notably, however, both quantitative and qualitative urine GAG tests can be insensitive, particularly if the urine is dilute, and cannot rule out Sanfilippo syndrome owing to the significant rate of false-negatives [31–37]. Therefore, in cases of increased clinical suspicion with a negative urine GAG screen, follow-up with enzyme analysis or genetic testing is recommended. Semi-quantitative urine screening assays using cationic dyes on filter paper (eg the Berry spot test) have relatively high rates of false-positives and false-negatives and should no longer be used [30].

The analysis of GAGs is being replaced by the analysis of specific GAG species (eg heparan sulfate) using tandem mass spectrometry because of increased sensitivity and specificity to these species [38]. Tandem mass spectrometry is now routinely used in some laboratories and should become the predominant strategy for GAG analysis in the future.

#### Enzyme analysis

As above, Sanfilippo syndrome is caused by deficiencies in one of four enzymes that are associated with a defect in heparan sulfate metabolism [39–42]. Enzymatic analysis using blood leukocytes or cultured fibroblasts is the recommended gold standard for confirmation of diagnosis of Sanfilippo syndrome and can be considered as a first-line test, particularly when there are difficulties obtaining a suitable urine sample and/or shipping in adequate conditions (ie < 4 °C, delivered within 24 h) [43]. At the very least, enzyme analysis should be performed in patients with increased GAGs (heparan sulfate) or if clinical suspicion is increased [43]. Enzyme activity and the presence of heparan sulfate fragments can also be measured by mass spectroscopy in dried blood spots (DBS), which offers considerable practical advantages (eg sample collection, storage, and transport), and multiple enzyme activity tests can be performed on a single sample [44, 45]. In addition, parameters such as sample viscosity, hematocrit level, and contamination during the drying process can affect the sensitivity, reproducibility, and overall accuracy of DBS measurement [46]. Therefore, a deficient enzyme result in DBS samples should be confirmed by an enzyme assay in leukocytes or fibroblasts, and/or by molecular genetic analyses [31]. False-negatives have not been reported in newborn screening pilot studies with this methodology, but clinical suspicion beyond the newborn period should always trigger laboratory testing.

Multiplex tandem mass spectrometry provides the potential to assay all enzymes simultaneously via high-throughput screening [47]. Alternatively, each enzyme can be assessed individually and ordered in a sequence according to the relative frequency of disease subtypes in the region; however, this assay is labor intensive and good quality control is essential [43]. The clinical features of Sanfilippo syndrome are similar to other conditions, such as multiple sulfatase deficiency and mucolipidosis. Therefore, if the sulfatase enzymes for Sanfilippo syndrome type A (heparan-*N*-sulfatase) or type D (N-acetylglucosamine 6-sulfatase) are deficient, then at least one other sulfatase should be assayed to rule out multiple sulfatase deficiency [31]. Conversely, if multiple lysosomal enzymes are elevated, the diagnosis of mucolipidosis should be suspected and confirmed by DNA testing.

#### Molecular genetic testing

A suspected diagnosis of Sanfilippo syndrome can be confirmed by molecular genetic testing or mutation analysis [31]. Molecular genetic testing should be offered to all patients as it enables cascade molecular screening of undiagnosed siblings or extended family members and family members who are carriers [31], thereby enabling appropriate genetic counseling and informed family planning [30]. In addition, the findings of molecular testing may inform clinical expectations of disease progression according to the pathogenicity of the mutation and knowledge regarding the correlation of genotype with phenotype [30]. Molecular testing results may also impact the patient’s eligibility for clinical trials and future therapies.

In instances where a patient’s primary diagnosis is made based on a molecular genetic diagnosis, a confirmatory biochemical assay should be conducted to confirm the pathogenicity of the mutations [48, 49]. When a patient is identified as homozygous for a mutation that is pathogenic for Sanfilippo syndrome or heterozygous for two known pathogenic mutations, a diagnosis can be made with reasonable confidence if the patient has a clinical phenotype consistent with Sanfilippo syndrome.

#### Prenatal diagnosis

Prenatal diagnosis is feasible for Sanfilippo syndrome in the context of a known familial diagnosis. The main methods used to collect material for prenatal testing are amniocentesis and chorionic villus sampling, which enable biochemical and molecular testing of fetal-derived tissues. If there is an older sibling with a confirmed diagnosis of Sanfilippo syndrome who has two known mutations, prenatal diagnosis may be made with molecular testing alone [1, 27, 44].

#### Newborn screening

Newborn screening provides the opportunity to diagnose patients as early as possible and enable prompt intervention with optimal outcomes when disease-specific therapies are approved [31]. Given the progressive and seemingly irreversible nature of the neurological manifestations of Sanfilippo syndrome, the adoption of all available measures to detect patients as early as possible should become standard practice. Most newborns with Sanfilippo syndrome are asymptomatic at birth; therefore, the identification of biochemical or genetic markers of Sanfilippo syndrome in newborns is crucial [50].

A suitable method for the screening of newborns is one that is rapid, cost-effective, sensitive, and widely available [50]. Newborn screening for MPS disorders has been studied with several methods, including GAG assay in urine, GAG assay in DBS with ultra-performance liquid chromatography combined with tandem mass spectrometry, fluorometric enzyme assay, digital microfluidics enzyme assay, and enzyme and/or substrate assay with tandem mass spectrometry (MS/MS) [50]. Overall, the recommended approach for the screening of MPS disorders is the analysis of enzyme activity in DBS with MS/MS or fluorometry to identify MPS subtypes, which is not possible with GAG assays. Newborn screening with molecular genetics tools is being considered; however, these tools are less readily available compared with biochemical testing [50].

In countries with newborn screening facilities, Sanfilippo syndrome is generally not included in routine screening programs; however, pilot studies for Sanfilippo syndrome types A and B are in progress. As disease-specific therapies become available and improve the lives of patients with Sanfilippo syndrome, the ethical and clinical imperative for early (pre-symptomatic) diagnosis will strengthen. Moreover, although newborn screening cannot determine disease severity, such programs provide timely information that may inform planning for families, even prior to the availability of commercially approved treatments.

### General principles and goals of management

In the absence of a disease-modifying treatment for Sanfilippo syndrome, the primary goal of management should be to optimize the quality of life for patients and their families. This requires a holistic approach that considers the wide-ranging and complex medical needs of patients with this condition. A key step in this process is the establishment of a multidisciplinary team of healthcare professionals to work collaboratively and in partnership with patients with Sanfilippo syndrome and their families. This multidisciplinary team would include (but is not limited) to physicians, nurses, therapists (eg physical, occupational, and speech), dieticians, psychologists, social workers, special educators, and counselors. A supervising clinician should oversee the coordination of care. Comprehensive care should be initiated as early as possible, ideally immediately after diagnosis, and the frequency of clinic visits and assessments should be tailored to meet the individual needs of each patient with Sanfilippo syndrome and their family. Frequent communication with families is important to align on care goals and plans, and to ensure that the best interests and values of patients and their families remain at the heart of the decision-making process.

Different assessments and interventions are required for patients with Sanfilippo syndrome depending on their level of disease progression (Table 3), and treatment plans should be modified according to each patient’s needs. For example, during the pre- and early symptomatic time frames, it is important to establish a multidisciplinary care team, initiate supportive care measures, conduct forward planning with families around future care needs, and provide genetic counseling as a part of family planning. As a patient’s disease progresses, supportive care measures need to be increased to alleviate the burden of symptoms and to support engagement in everyday activities as much as possible. For patients showing signs of pain, distress or behavioral changes of undetermined etiology, systemic assessments of likely causes of pain are recommended (Table 4). In the later stages of the disease, the maintenance of quality of life and prevention of complications become the priorities of care.

**Table 3:** Key evaluations for the monitoring of Sanfilippo syndrome at diagnosis and throughout the disease course*

Area of assessment	At diagnosis	Regularly	As clinically indicated
Neurodevelopment/neurological	• Cognitive function (formal evaluation) • Adaptive behavior skills (formal evaluation with VABS) • Gross motor function • Fine motor skills • Tone • Sleep • Seizure activity • Movement (walking/gait) • Behavioral symptoms • High-resolution MRI	• Every 6–12 months (by physical exam/history and/or formal evaluation): • Gross motor • Fine motor • Tone • Sleep • Seizure activity • Movement (walking/gait) • Behavioral changes	• High-resolution MRI (triggered by extreme behavioral changes, unexplained pain or distress, suspicion of headaches, suspicion of elevated intracranial pressure, sudden neurological or functional declines) • Evaluation for behavior-based therapy
Seizures			• EEG (triggered by suspected seizure activity; see the seizure management section)
ENT	• ENT examination • Audiologic testing	• At least every 12 months: • ENT examination • Audiologic testing	• ENT examination and audiologic testing: • Triggered by recurrent otitis media or suspected changes in hearing • At least 6-monthly if identified hearing loss or otitis media with effusion • Flexible endoscopy prior to general anesthesia: • Triggered by suspicion of airway obstruction
Airway/respiratory	• Vital signs • Respiratory examination		• Sleep evaluation (triggered by sleep disturbance) • Medical workup (triggered by sleep disturbance, recurrent pneumonia, impaired secretion management)
Surgery			• Pre-operative assessment: anesthetic review, airway assessment, cardiology review, respiratory review, hematology review, neurologic review, palliative care, and nursing review
Ophthalmology	• Full ophthalmologic evaluation	• Every 12 months: • Full ophthalmologic evaluation with dilation	• Full ophthalmologic evaluation (triggered by persistent unexplained pain, distress or agitation, falls) • Electroretinogram (triggered by suspicion of retinopathy)
Dental		• Dental exam at least every 6 months, or every 12 months if sedation is required	• Dental exam (triggered by persistent unexplained pain, distress or agitation)
Nutrition and gastroenterology	• Assessment of eating, drinking, and swallowing abilities • Electrolytes and liver function tests	• At least every 12 months: • Assessment of eating, drinking, and swallowing abilities • Electrolytes and liver function tests	• Monitor for gastroesophageal reflux (triggered by increased behavioral distress, sleep disturbance, and/or other clinical signs) • Diet assessment (triggered by weight loss or poor growth) • Abdominal imaging (triggered by persistent unexplained pain, distress or agitation)
Cardiac	• Echocardiogram • ECG	• Every 12 months: • ECG • Every 24 months: • Echocardiogram	• Echocardiogram (at least 12-monthly if abnormalities on initial or subsequent assessments) • Holter monitoring (triggered by abnormal ECG)
Orthopedic	• Physical exam • Scoliosis series X-ray • Bilateral hip X-ray • Full spine films • Range of motion (upper and lower extremities)	• Every 6 months: • Range of motion • Every 1–2 years from age 7 years: • Physical exam • X-rays (scoliosis and bilateral hip) • Monitor trigger finger, genu valgus deformity, femoral anteversion, tibial torsion • Use of established measurement tools to monitor trajectory of motor skills and subsequent needs	• Physical exam and X-rays (scoliosis and bilateral hip; triggered by rapid progression of orthopedic manifestations or unexplained signs of discomfort or pain) • Serum vitamin D level (in patients with impaired mobility) • Bone mineral density (in patients with prolonged functional immobility, for whom there is a concern of fracture risk)
Pain		• Standardized pain assessments • Caregiver proxy assessments	• Medical workup to investigate etiology (see Table 4)
Hematology			• Complete blood count with differential (triggered by persistent unexplained pain, distress or agitation, or unusual and/or prolonged bleeding) • Prothrombin time, partial thromboplastin time, and complete blood count prior to invasive procedures (if not done in the preceding month)
Occupational therapy***	• Evaluate and support fine motor skills**	• Every 6 months: • Supportive equipment needs	• Ongoing monitoring through therapeutic sessions to adapt therapeutic strategies and supports**
Physical therapy***	• Evaluate and support fine motor skills** • Range of motion in upper and lower extremities	• Every 6 months: • Range of motion in upper and lower extremities • Supportive equipment needs	• Ongoing monitoring through therapeutic sessions to adapt therapeutic strategies and supports**
Speech therapy***	• Evaluate and support communication and eating/drinking/swallowing skills**	• Speech and language skills • Evaluate need for AAC devices and strategies	• Ongoing monitoring through therapeutic sessions to adapt therapeutic strategies and supports** • AAC strategies should be implemented as early as possible prior to loss of verbal speech
Growth		• Growth parameters (height, weight, and head circumference) measured at routine visits and plotted on Sanfilippo syndrome-specific growth curves [122, 123]	
Puberty		• Monitor pubertal development	• Referral to pediatric endocrinology (triggered by premature pubertal development noted on exam)
Family support	• Counseling	• Counseling • Assessment of anxiety, depression, and chronic traumatic stress • Service needs such as respite care, caregiving support, government social program and benefit referrals, and connections to disease patient advocacy groups**	

* Clinical judgment may be used to determine if deviation from the above schedule is appropriate based on the patient’s clinical history, extent of organ manifestations, variability of disease phenotype, and in collaboration with the family as to the potential burden of assessments

**Indicates areas where the steering committee of clinical experts have added to the content derived from consensus guideline statements

***Rehabilitative therapy evaluation tools may be chosen by the local clinician based on availability and which instrument is best suited for the individual patient

AAC, augmentative and alternative communication; EEG, electroencephalography; ENT, ear nose, and throat; MRI, magnetic resonance imaging

**Table 4:** Key evaluations for patients in pain, distress, or with behavioral changes of undetermined etiology

Area of assessment	Evaluations
Neurodevelopment/ neurological	High-resolution MRI: assessing for causes of headaches, signs of raised intracranial pressure and/or other intermittent or acute abnormalities that could be a cause of pain, distress, or behavioral changes
ENT	ENT examination: assess for potential causes of unexplained pain, including infection
Ophthalmology	Full ophthalmologic evaluation: assess for potential causes of unexplained pain, distress, agitation, or falls
Dental	Dental exam: assess for potential causes of unexplained pain, distress, or agitation
Nutrition and gastroenterology	Assess for gastroesophageal reflux as potential cause of behavioral distress and/or sleep disturbance Abdominal imaging: assess for potential causes of unexplained pain, distress, or agitation
Orthopedic	Physical exam and X-rays: assess for potential causes of unexplained signs of discomfort or pain, particularly hip disease
Pain	Standardized pain assessments Caregiver proxy assessments
Laboratory investigations	Complete blood count, electrolytes, serum chemistries, and urine analysis
Detailed physical exam and history	Exam and history to include areas described above, as well as skin and genitourinary evaluation (including assessment for urinary retention)*

*Indicates areas where the steering committee of clinical experts have added to the content derived from consensus guideline statements

ENT, ear, nose, and throat; MRI, magnetic resonance imaging

In addition to their impact on the patient, neurodegenerative conditions such as Sanfilippo syndrome can have a strong negative impact on the psychosocial functioning and quality of life of family members [51–55]. Parents and caregivers face potentially traumatic medical events followed by short- and long-term stress [56], putting them at risk of developing parental post-traumatic stress disorder (PTSD) [56–58]. For example, 22% of parents of children with Sanfilippo syndrome in the Netherlands were found to be suffering from PTSD, compared with 3.8% of parents in the general population of the same country [59]. The presence of parental PTSD can, in turn, have a significant influence on the psychological wellbeing of the affected child [60]. Thus, the adoption of a trauma-informed approach to caring and supporting families affected by Sanfilippo syndrome is imperative [61].

### Managing the neurological challenges of Sanfilippo syndrome

#### Monitoring of neurodevelopment

Progressive CNS degeneration is a characteristic feature in patients with Sanfilippo syndrome, with neurological plateau and eventual regression following initial normative gains in neurodevelopment [1, 62]. Clinical heterogeneity exists between and within the four disease subtypes and the rate of neurocognitive decline varies. While broad genotype–phenotype correlations have been recognized in some cases [15], these are not universal [1, 62]. Patients should therefore undergo detailed neurological evaluation at diagnosis and regular monitoring (eg every 6–12 months) thereafter to detect changes in cognition, motor function, and behavior.

The most well-characterized neurocognitive phenotypes are Sanfilippo subtypes A and B. In these forms of the disease, patients generally continue to acquire cognitive skills until the age of 2.5–4 years depending on the subtype and severity phenotype [62]. Data for Sanfilippo syndrome types C and D are limited [63]. However, depending on phenotype, the timing of developmental plateau and pace of decline can vary. Speech and language delay are the most frequent initial symptoms, and language delay may be apparent by the age of 2 years, before cognitive decline starts [21, 64]. Conductive hearing loss secondary to middle ear disease is commonly comorbid, along with the development of high frequency sensorineural hearing loss [21], and further impacts the acquisition of critical early language skills. Indeed, in this context, the effective management of middle ear disease and sensorineural hearing loss typically results in significant improvements. Monitoring of neurocognitive function is recommended on an ongoing basis (or at a frequency appropriate to each individual’s needs) to help families identify areas of strength and interval loss of skills. In addition, this monitoring will help to support discussions focused on helping families adjust to progression of the disorder, educational needs, and supportive interventions in the later phases of the disease. There are many psychometric measures that can be used to evaluate cognitive function in patients with Sanfilippo syndrome [65]. The Bayley Scales of Infant and Toddler Development, Third Edition (Bayley-III) is one of the most frequently used in clinical studies [62]. However, use of a specific instrument for clinical care purposes did not reach consensus in our survey. Clinicians may use an available tool that is best suited for monitoring their individual patient over time; recognizing that use of measures that align with published studies may allow for a more informed comparison of the patient’s results with existing disease natural history data.

In addition to assessing neurocognitive function, magnetic resonance imaging (MRI) of the brain should be conducted at baseline and as clinically indicated. Neurodegeneration in patients with Sanfilippo syndrome can be represented by decreases in the volume of cortical and subcortical parenchyma, with secondary increases in ventricular volume on MRI over time [63]. These changes occur in parallel with cognitive decline and are much more severe in patients with rapidly progressing phenotypes than in those with slowly progressing phenotypes [64]. Triggers for ordering an MRI of the brain, beyond the baseline assessment, may include extreme behavioral changes, unexplained pain or distress, suspicion of headaches, suspicion of elevated intracranial pressure, and sudden neurological or functional declines. Opportunistic neuroimaging may also be considered during anesthesia for another reason, provided that the risks and benefits are weighed and discussed with the family.

#### Motor function

Assessment of gross motor and fine motor function is recommended at diagnosis of Sanfilippo syndrome and then every 6–12 months, or more frequently if clinically indicated. Fine motor skills reach a plateau at approximately 2–3 years of age in patients with Sanfilippo syndrome types A and B with typical progression of the disorder (mirroring cognitive decline), whereas development of gross motor skills tends to be preserved until later. In this regard, one study of patients with Sanfilippo syndrome types A, B, and C reported the onset of clumsiness in walking at a median age of 7 years, 7.5 years, and 9 years, respectively, and loss of unassisted sitting at 10.5 years, 14 years, and 13.5 years, respectively [22]. Given cognitive impairment and hearing loss, difficulties in following instructions together with poor imitative skills may impair the ability of patients with Sanfilippo syndrome to perform motor tasks after this age.

Walking and gait should be assessed at baseline then every 6–12 months, or as needed. Clinicians should be particularly mindful of functional impairments and the development of movement disorders such as dystonia, ataxia, and dyskinesias (including tics, myoclonus, and choreoathetosis). As the disease progresses, patients may require more time to initiate or complete a task owing to the development of motor apraxia and challenges with motor planning. This additional time should be accommodated in clinical exams, formal testing, and during educational and therapeutic activities. Consideration should be given to the needs for medical equipment such as orthotics and bracing, and when to refer to orthopedics, physiotherapy, or other supportive care functions.

#### Considerations for neurobehavioral, psychological, and psychiatric care

Changes in neurocognition at 2–4 years of age typically coincide with the appearance of behavioral difficulties, including hyperactivity, hyperorality and/or preservative chewing, temper tantrums, disobedience or unresponsiveness to discipline, decreased attention, and severe sleep disturbance [4, 8, 10, 19, 66–68]. Most patients with Sanfilippo syndrome develop autistic-like behaviors (primarily social and emotional abnormalities from approximately 4 years of age [25, 69]), and as cognitive function declines, many patients display disinhibited behaviors [70]. For patients with slowly progressing disease who survive into adulthood, reported behavioral problems include motor restlessness, screaming, sensitivity to touch or temperature changes, anxiety, crying fits, aggressive behavior, stereotypic speech, and irritability [19]. Another report found that adults with Sanfilippo syndrome tend to engage less in interactions and become withdrawn [9].

The management of behavioral symptoms requires a holistic approach of understanding the behavior in the context of the cognitive skill level, creating a safe environment at home and school for the patient, and providing a routine and structure in addition to any pharmacologic approaches [9, 71]. Developmental testing should be conducted in an environment familiar to the patient by a tester who has an established rapport with the patient and has familiarized themselves with the behavioral characteristics of Sanfilippo syndrome prior to testing. When evaluating and monitoring adaptive behavior skills, the Vineland Adaptive Behavior Scale should be used as at least one of the measures [72–74]. When considering behavior-modifying medications, careful consideration of the following is needed to formulate a proper treatment strategy: any physical problem (eg pain), musculoskeletal problems, gastrointestinal disturbances, seizures, dental problems, and communication challenges.

The identification of negative stimuli (eg pain, an unfamiliar situation or environment, or the association of an unpleasant feeling with a specific location) is needed to prevent or mitigate abnormal behavior [9, 71]. The input of parents and caregivers should be encouraged to help calm and comfort the patient when completing necessary medical tests and exams. If necessary, tests may be conducted under anesthesia when coordinated with other procedures.

Early identification of behavioral changes and sleep problems will help to enable effective management and referral to appropriate specialized services [4, 15]. Regular neurologic assessments are recommended at baseline and then every 6–12 months, and more frequently if clinically indicated [18]. Evaluations should monitor the appearance or changes in sleep disturbances, seizure activity, neuromuscular tone, movement disorders, and behavior.

The identification of psychiatric symptom clusters in the context of the developmental age-equivalent profile of each patient is helpful when considering interventions to manage the neurobehavioral aspects of Sanfilippo syndrome. These clusters include sleep, ADHD and autistic behaviors, social communication difficulties, speech and language difficulties, sensory difficulties, and anxiety. Applied behavioral analysis therapy, where available and tailored to the individual patient, should be supported to enhance communication skills, maintain motor abilities, reduce unsafe behaviors, and reduce behaviors that interfere with learning and engagement as it has been found to be beneficial for some patients with Sanfilippo syndrome [75].

Several groups of behavior-modifying medications have been administered to patients with MPS disorders; however, published evidence for the use and long-term effectiveness of these agents in patients with Sanfilippo syndrome is limited [9]. Therefore, the prescription of psychiatric medication targeted to mitigate behavioral symptoms should be accompanied by an evaluation of contraindicated risks. Such evaluation is particularly important given that Sanfilippo syndrome is a multisystem disease and the impact of psychotropic medication on cardiac, hepatic, and renal systems needs to be taken into account. Use of stimulant medications, mood stabilizers, antipsychotics, and antianxiety drugs may be considered on a case-by-case basis and with short-term trial periods following a review of the potential risks and benefits with the patient’s family.

### Seizure management

Seizures have been reported in patients with MPS disorders in which GAG accumulation in the brain is speculated to trigger alterations in neuronal connectivity and signaling, and release of inflammatory mediators [4, 76]. Approximately 26–52% of patients with Sanfilippo syndrome will develop seizures and epilepsy in the later stages of the disease [4, 14, 15, 68, 76]. While the prevalence of seizures does not differ greatly between patients with the four subtypes of Sanfilippo syndrome, the age of onset of seizures appears to be somewhat earlier in patients with type A than in those with other subtypes [4, 14, 15, 17, 19, 22, 77], and the incidence of seizures has been found to increase with advancing neurocognitive deterioration [4].

Patients with Sanfilippo syndrome typically present with generalized tonic–clonic seizures [19, 22, 78, 79]. A study of electroencephalography (EEG) records of patients at different stages of Sanfilippo syndrome found that progressive EEG changes correlated with age and disease progression [80]. While patients younger than 3 years had normal background activity while awake, slowing of occipital-dominant rhythm and background activity at wakefulness could be observed after 6 years of age and became more severe after 11 years of age. Non-convulsive status also was noted in a couple of patients [80]. EEG abnormalities during sleep have also been reported [79]. Nocturnal seizures can disrupt sleep hygiene, which in turn can exacerbate and contribute to diurnal somnolence, disturbed concentration, and neurobehavioral lability [76].

Optimal management of patients with epileptic seizures requires a correct diagnosis. Clinicians should have a high index of suspicion in monitoring for epileptic activity (convulsive and non-convulsive) in patients with Sanfilippo syndrome. However, seizures can be difficult to detect in patients with Sanfilippo syndrome as they often become evident by alterations and/or abnormalities in mental status, behavior, and/or cognition, which are inherent features of the disease [81, 82]. The occurrence of absence seizures and non-convulsive *status epilepticus* can be subtle and difficult to monitor. A diagnostic workup for seizures in patients with Sanfilippo syndrome should include electrophysiological examination by EEG, and prolonged video EEG or in-home mobile EEG may be warranted to detect more subtle seizure activity and nocturnal seizures.

Both convulsive and non-convulsive epilepsy should be adequately treated according to the patient's individual needs and medication history. Literature discussing the treatment of epileptic seizures in patients with Sanfilippo syndrome is limited. Anecdotal evidence based on the experience of experts in the treatment of epilepsy in patients with MPS disorders indicates there are not clinically significant differences in seizure control and management between patients with Sanfilippo syndrome and other patients with epilepsy [76]. Therefore, standard protocols for the treatment of seizures should be followed [53]. Preference should be given to anti-epileptic drugs with fewer drug–drug interactions that do not require the monitoring of therapeutic drug levels.

### Sleep

Sleep alterations are an almost constant feature of Sanfilippo syndrome, affecting 87–92% of patients [67, 83]. Features include difficulties with settling, frequent nocturnal waking and wandering, and greater daytime sleep compared with healthy individuals [2, 10, 84]. The unrelenting nature of sleep disturbances places a heavy burden on both the patient and their family, and can cause great distress [10, 84].

In patients with sleep disturbance, medical workup should include consideration of the presence of disordered movement or seizure activity [85], iron deficiency in the setting of restless legs, pain or intercurrent illness, esophageal reflux, dental disease, and disordered breathing or sleep apnea during sleep. Sleep disturbance should be addressed with a multimodal approach that includes sleep hygiene counseling, implementing behavioral strategies, addressing the safety of the environment (eg securing the door to prevent harm from wandering, removing items that may cause choking, removing or covering hard surfaces, enclosed specialty beds, and avoiding furniture that could be toppled over), treating circadian rhythm disturbance, and other comorbid medical factors. The use of sleep diaries is encouraged for monitoring changes, evolution of sleep disturbance, and response to interventions.

Sleep apnea is well described as a cause of sleep disturbance in patients with MPS disorders [86, 87]. A history of sleep apnea and snoring should be sought in all patients with Sanfilippo syndrome who also have sleep disturbance [87], and diagnosis and management of sleep apnea should be made under the guidance of a pulmonologist and/or otolaryngologist depending on etiology. If the patient has signs and symptoms of obstructive sleep apnea along with adenoid and/or tonsillar hypertrophy, removal of adenoids and/or tonsils should be performed without delay. This may need to be repeated if tissue regrowth and obstructive sleep apnea recurs later. Continuous positive airway pressure (CPAP) therapy should be considered for patients who display the presence of obstructive sleep apnea that persists after adenoidectomy and/or tonsillectomy. The implementation of CPAP will likely require additional longer-term behavioral or other supports to help increase the patient’s acceptance of the device. The ongoing follow-up of patients who are receiving medication for respiratory and sleep disorders is recommended, the frequency of which will depend on the severity of the respiratory disease and sleep-disordered breathing.

Patients with Sanfilippo syndrome may experience disruption of their circadian rhythm [86, 88], which may be partly addressed by melatonin supplementation [9, 10, 71]. If melatonin is started, it is recommended to begin at a low dose (0.5–2 mg) and then be titrated up to higher doses per the patient’s response [10, 89]. The typically recommended dose is 2–10 mg at bedtime, but a higher dose is occasionally needed.

### Managing the airway

#### Respiratory management

Respiratory tract and sinopulmonary infections are common in patients with Sanfilippo syndrome [12], and respiratory complications such as pneumonia have been reported as the primary cause of death in these patients [90]. However, behavioral disturbances in patients with Sanfilippo syndrome may mask the typical signs of respiratory infection, leading to diagnosis after the respiratory infection has advanced. Therefore, clinicians should consider a diagnosis of pneumonia in patients with Sanfilippo syndrome and should order early diagnostic radiology when a respiratory infection is suspected, with prompt treatment with antibiotics when pneumonia is confirmed.

As part of routine clinical care, patients with Sanfilippo syndrome should undergo regular clinical assessments and physical examination to facilitate the early detection of respiratory or other complications. These steps should include assessment of vital signs (eg respiratory rate, heart rate, height, and weight) and routine respiratory examination (including the nose and oropharynx). Investigation of clinical history should include sleep hygiene, quality and duration, symptoms of sleep-disordered breathing, history of respiratory symptoms (eg chronic cough), history of pneumonia, history of oral secretions, history of difficulty feeding, history of gastroesophageal disease, and history of nasal secretions and/or nasal congestion. Abnormal findings may prompt measurement of oxygen saturation and non-invasive monitoring of carbon dioxide, if available. Excessive oral secretions may be managed by manual suction and/or medications such as atropine or glycopyrrolate [84, 91, 92].

Routine childhood vaccinations should be given per the standard of care schedule, including annual influenza vaccines. Pneumovax 23 is recommended for patients with Sanfilippo syndrome, in accordance with the guidelines for those who are at increased risk of pneumococcal disease [12]. While the potential increased risk of serious illness owing to COVID-19 infection in patients with Sanfilippo syndrome is considered likely, there is limited experience in these patients and vaccination is recommended in line with accepted global protocols.

#### Anesthesia and peri-operative care

Patients with Sanfilippo syndrome may require anesthesia for surgical interventions (eg dental extractions and tonsillectomy) to help manage their disease or to carry out evaluations such as MRI, lumbar puncture, or echocardiography [93, 94]. Complications during anesthesia and surgery can occur in patients with Sanfilippo syndrome [94, 95], albeit typically at a lower rate than in patients with other MPS disorders [96]. A retrospective analysis of 126 cases of anesthesia in 37 patients with Sanfilippo syndrome found that the most common anesthesia-related complications were bradycardia or tachycardia (2.4% of anesthesia events), respiratory insufficiency (1.6%), hypoxemia (1.6%), and atelectasis (1.6%) [96].

To mitigate the respiratory risks in patients with Sanfilippo syndrome, sedation and anesthesia events should always be conducted in the hospital setting with experienced anesthesia personnel available and ready to manage complex airway emergencies. In situations where a procedure or evaluation would be most efficiently and humanely conducted under anesthesia, the number of such anesthesia events should be minimized within reason by combining procedures and coordinating efforts with the multidisciplinary team, as much as possible.

For patients with behavioral and cognitive challenges, patient-centered accommodations should be considered to ensure their safety and wellbeing before and after anesthesia [93–95, 97]. Such accommodations may include: allowing parent/caregiver access to the patient during the induction of anesthesia and upon emergence/recovery; providing a low-stimulus environment with the ability to secure/close doors to reduce the risk of the patient escaping; the use of distraction techniques and items; consideration of safety concerns with regards to impulsivity, hyperactivity, and flight risk; and provision for additional staff to supervise as appropriate to meet the needs of each patient.

Prior to anesthesia, nursing and medical providers should review advanced directives, baseline pain, and comfort care needs with the patient and their family. Pre-operative anesthetic review and airway assessment should be conducted prior to the day of the scheduled procedure to allow time to have any necessary equipment and staff available for the sedation event. Unless contraindicated, chronic medications should be given on the day of anesthesia within the confines of fasting guidelines, particularly anticonvulsants and neurobehavioral medications.

While anesthesia-related airway issues are less common in Sanfilippo syndrome than in other mucopolysaccharidoses, when they do occur, they can be serious.

When preparing for anesthesia in a patient with Sanfilippo syndrome, providers should be prepared for a potentially difficult laryngoscopy and intubation [93–95, 97]. If an upper airway obstruction which may complicate intubation is suspected, a pre-operative flexible endoscopy (nasopharyngolaryngoscopy) is recommended in order to inspect the upper airway. Laryngeal mask airway is a good alternative to tracheal intubation for many patients in whom a native airway is not feasible or if the procedure is short and non-invasive (eg MRI scan). General anesthesia with a native airway (without pharyngeal or laryngeal intubation) may be considered for patients with Sanfilippo syndrome; however, the use of standard airway maneuvers (eg chin lift, shoulder roll, CPAP, and oral or nasal airways) and adjuncts (eg CPAP) may be needed [93–95, 97].

### Somatic manifestations of MPS III

#### ENT and audiology considerations

Hearing loss is common in patients with Sanfilippo syndrome and can contribute to speech delay and behavioral and learning problems [9]. Hearing loss can be conductive, sensorineural, or mixed due to a combination of dysostosis of the ossicles of the middle ear, inner ear abnormalities, and frequent otitis media and impaired neurological function [12]. To ensure early detection, ENT examination and audiologic testing should be conducted immediately after diagnosis, with a follow-up at least every 12 months and more frequently if there are recurrent episodes of otitis or suspected changes in hearing. When there is identified hearing loss or otitis media with effusion, follow-up may need to be more frequent based on the individual patient.

When detected, the early and aggressive management of hearing impairment and ear effusion should be performed to optimize language development during critical developmental windows. ENT surgery remains a fundamental therapeutic procedure for reducing the frequency and severity of ear infections, even if the interventions are not curative [86, 98]. If a conductive type of hearing loss is detected owing to effusion in the ear (lasting more than 2 months bilaterally or 4 months unilaterally), grommets (ventilation tubes) should be inserted without delay to maximize hearing and reduce symptoms.

Audiology evaluation should include assessments of both air and bone conduction. Where hearing assessment is needed, and behavioral testing is not possible, auditory brainstem response testing under sedation or general anesthesia should be considered. Decisions on the use of hearing devices should be made in close collaboration with the family, and the hearing needs of the patient should always be clearly documented in their records and care plans with accompanying advice on communication and hearing supports, particularly in the educational setting. Standard ‘behind the ear’ hearing aids should be considered for patients with hearing loss. Challenging behavior should not be used as an excuse to dismiss a trial of hearing aids, particularly in the educational setting.

Ear disease may also present with an impairment in balance. Balance problems can significantly impact quality of life, particularly mobility, and may be overlooked in patients who are unable to communicate their symptoms effectively. Considering ear disease as a factor in emerging or worsening balance problems may uncover a potentially treatable etiology. ENT physicians can aid in specialized evaluation of these concerns.

#### Ophthalmologic considerations

A proportion of patients with Sanfilippo syndrome have affected eyesight, though the timing and progression of visual impairment has not been well studied to date. Pigmentary retinopathy is considered a prominent ocular manifestation in patients with Sanfilippo syndrome [29], with severity ranging from subclinical electroretinography features to moderate-to-severe clinical disease that leads to problems such as nyctalopia (night blindness) and overall decreased vision [99–102]. The corneas of patients with Sanfilippo syndrome type A and type B appear clear but have increased mean fibril diameter and fibril spacing [101, 103]. Optic atrophy and disk swelling have also been reported [104].

Routine ophthalmologic examination is recommended every 12 months and more frequently if clinically indicated. Ophthalmologic assessment should include assessment of vision in both eyes, orthoptic assessment, refraction, examination of anterior and posterior segment of the eye (including examination of the cornea, retina, and optic nerve), and measurement of intraocular pressure. Patients with behavioral challenges may require examination under anesthesia, in which case the risks and benefits must be weighed.

Given that clinical signs of vision loss may be difficult to detect or absent in patients with impaired communication, input from caregivers is essential. An electroretinogram can confirm the diagnosis when retinopathy is suspected owing to symptoms of night blindness or impaired vision in low light, visual field loss or reduction in vision, or signs of pigmentary retinal change, but the benefits of knowing versus the risk of anesthesia must be weighed.

Patients with Sanfilippo syndrome and visual impairment should be provided with access to low-vision supports and services in the home, community, and educational settings. Support for vision impairment should be included in educational settings as part of their individualized educational plan.

#### Dental care

The dental features of patients with Sanfilippo syndrome are not well described compared with those of other MPS disorders [105], with disease-specific observations limited to generalized obliteration of pulp chambers and root canals [106, 107]. However, patients with MPS disorders are typically considered at high risk of dental disease [105]. Therefore, basic good oral hygiene is recommended with twice-daily brushing and avoidance of drinking sugary beverages on a regular basis.

Regular dental visits, preventive fluoride applications, and dental treatment must be included in the multidisciplinary team approach. Oral health problems should be ruled out in the setting of behavioral changes, agitation, distress, changes in sleep patterns, changes in eating habits, or a change in oral sensory behaviors.

In patients who have challenges clearing food from the oral cavity or who take daily sweetened liquid medications, water should be offered or teeth wiped after meals and snacks. As brushing the teeth can be challenging in patients who have a sensory aversion to or do not understand this task, supports such as three-sided toothbrushes, bite blocks, and distraction techniques may be helpful. Dental sealants are recommended to prevent and/or arrest dental caries in primary and/or permanent molars, and they should be monitored for integrity at each dental visit and restored as indicated. If sedation is required for dental procedures, dental care should take place in a tertiary care facility with experienced anesthesia staff.

#### Nutritional and gastrointestinal management

Gastrointestinal disturbances are common in patients with Sanfilippo syndrome and typically include chronic or recurrent non-infectious loose stools and/or constipation [12]. Stooling issues can be a source of discomfort and distress for patients, which may manifest through an increase in behavioral disturbances, heightened sleep disturbance, or other alternative expressions of pain. To mitigate discomfort and distress, therapeutic maintenance regimens should aim for consistent and adequate stool elimination to maintain the comfort and health of the patient.

Diarrhea is typically episodic for patients with Sanfilippo syndrome but can be persistent in some individuals and can be exacerbated by frequent antibiotic treatment or recurrent infections. The management of diarrhea includes medications when needed (eg synthetic opioids to reduce gut motility). The care plan should note, particularly for all care providers in the educational and therapeutic settings, that non-infectious Sanfilippo syndrome-related diarrhea should not be a cause for exclusion from educational and therapeutic activities.

No specific dietary plan has been studied in Sanfilippo syndrome to guide dietary recommendations, outside of general advice for a healthy diet. Monitoring and restoration of micronutrient deficiencies is recommended to support metabolic functions. Patients should also be monitored for gastroesophageal reflux, which may contribute to increased behavioral distress or increased sleep disturbance. Where present, a trial of anti-reflux medication, diet modification, or a combination thereof should be considered.

Assessment of eating, drinking, and swallowing abilities should be performed by a speech–language–feeding therapist at diagnosis and then monitored at least yearly if clinically indicated. Steering committee clinicians further recommend that primary care providers elicit history regarding any safety concerns with eating, drinking, and swallowing routinely at scheduled visits, prompting further referral as needed. Clinical assessments provide the best information when conducted at mealtimes and in a variety of settings (eg home and school) to observe any accompanying behavioral and cognitive challenges around mealtimes.

Referral to a dietician is recommended for patients with Sanfilippo syndrome who have a substantially self-limited diet, experience weight loss or poor growth, have sensory needs limiting proper nutrition, or experience a decline of oromotor skills that impairs normal caloric intake within a reasonable time frame. Such referral should be made in conjunction with referral to or consultation with a speech–language–feeding specialist. Diet and fluid modifications should be made using the International Dysphagia Diet Standardization Initiative framework (https://iddsi.org/framework/) in consultation with a trained speech–language–feeding therapist. In instances of inadequate nutrition via oral feeding or presence of significant risk of aspiration or history of aspiration pneumonia, placement of an enteral feeding tube should be considered jointly with the patient’s family. When red flags are present for pharyngeal dysfunction (eg cough, wet voice, or recurrent lower respiratory tract infections), the patient should be referred for a Videofluoroscopic Swallowing Study in consultation with a speech–language–feeding therapist.

Other gastrointestinal manifestations of Sanfilippo syndrome include elevations in liver enzymes (alanine aminotransferase ≤ 3.5 times upper limit of normal [ULN]; aspartate aminotransferase ≤ 1.5 × ULN) and hepatomegaly, which do not typically require intervention. Hernias of the umbilicus and inguinal area should be monitored on routine examination and may require intervention if they become problematic.

#### Cardiac manifestations

In rare cases, cardiac manifestations may require intervention in patients with Sanfilippo syndrome. GAG accumulation can lead to cardiomyopathy, low-grade valve disease and/or dysplastic valves, arrhythmia owing to heparan sulfate accumulation in the conduction system, and other complications that may be problematic in patients surviving to adulthood [108, 109]. For example, at least two case studies describe adults with Sanfilippo syndrome types A and C presenting with symptomatic atrioventricular block that required implantation of a pacemaker [110, 111].

In a study of 30 patients with Sanfilippo syndrome (n = 16 aged < 18 years), none of the individuals had significant signs or symptoms of cardiac disease, but subclinical systolic and diastolic dysfunction and valvular abnormalities were prevalent, and about 16% had a first-degree atrioventricular block on electrocardiography (ECG) [108].

All individuals with Sanfilippo syndrome should have baseline cardiac evaluation at diagnosis to include a physical exam, vital signs (eg blood pressure), echocardiogram, and ECG. Thereafter, an echocardiogram is recommended every 24 months if no abnormalities are noted at the initial echocardiogram. If abnormalities are noted on initial or subsequent echocardiograms, the frequency should increase to every 12 months.

A 12-lead ECG and rhythm strip is recommended every 12 months in patients with Sanfilippo syndrome, and as needed owing to the difficulty of assessing symptoms in these patients. If an ECG is abnormal, a Holter monitor should be placed for at least 24–48 h for a more thorough evaluation.

#### Management of orthopedic complications

Orthopedic complications are a source of discomfort and distress in patients with Sanfilippo syndrome, often involving changes to the hips and spine [112]. Osteonecrosis of the femoral head and hip dysplasia can be a source of particularly severe discomfort, and intervention should be considered on a case-by-case basis. Complications requiring surgical intervention include progressive scoliosis [112]. Low bone mass and vitamin D insufficiency or deficiency are prevalent, and patients with decreased mobility or a history of receiving anti-epileptic medication are at risk for osteoporosis and fractures [113].

Patients with Sanfilippo syndrome should undergo a thorough orthopedic examination at the time of diagnosis. Consensus statements additionally endorse X-ray evaluation of the hips and spine at diagnosis and every 1–2 years from 7 years of age onwards, or sooner if clinically indicated. However, after thoughtful review, the expert clinician steering committee recommends further refinement of this guidance based on accessibility of specialists and procedural risks. It is felt that for patients without overt musculoskeletal symptoms, the initial orthopedic evaluation may be performed by the primary care clinician through a close musculoskeletal exam and a baseline X-ray of the hips and spine. Weighing the risks of cumulative radiation exposure with repeated monitoring X-rays, the steering committee suggests that radiography be performed at the time of diagnosis and then repeated only as clinically indicated. Unless there is clinical suspicion, monitoring for cervical spine instability is not recommended. In addition to the routine musculoskeletal exam, annual visits should also monitor for trigger finger, genu valgus deformity, abnormal spinal curvature, femoral anteversion, and tibial torsion that do not appear to improve or are worsening with age, with referral to an orthopedic specialist as clinically indicated. Given that pain may be difficult to assess and localize in patients with cognitive impairment and behavioral disturbances, radiographic studies of the hips should be considered in the evaluation of otherwise unexplained signs of discomfort or pain.

### Facilitating day-to-day activities and maintaining quality of life

#### Management of pain and distress

Although the neurological features of Sanfilippo syndrome may be the primary focus of patient care, physical manifestations such as pain and discomfort due to musculoskeletal or gastrointestinal problems can further exacerbate the neurocognitive and behavioral challenges experienced by individuals with this condition [9]. Thus, after appropriate evaluation for treatable medical complications, management of pain should be a fundamental part of the care of patients with Sanfilippo syndrome, with the aim of improving their quality of life and maintaining mobility. Notably, patients with cognitive impairment may express pain or discomfort via a range of behaviors that are non-classical and individual to them [114].

There should be a low threshold for investigation of sources of pain in patients with escalating abnormal behaviors or acutely worsening sleep disturbances, or signs of significantly increased agitation. Investigation for sources of pain or discomfort may include consideration of headaches (with consideration of alterations in intracranial pressure or symptoms of normal-pressure hydrocephalus), abdominal discomfort (eg acid reflux, ulcers, intestinal gas pain, and constipation), joint disease (eg arthralgia, arthritis, and osteonecrosis of femoral head), ENT issues (eg otitis and sinusitis), and dental-related pain (Table 4). In the case of persistent pain, distress, or agitation for which outpatient evaluation has been unrevealing, admission to hospital for thorough efficient medical workup is recommended. This workup should include hip and spine X-ray; dental examination for signs of decay; abdominal imaging to investigate potential constipation or other obstruction; eye exam with consideration for signs of elevated intracranial or intraocular pressure; complete blood count with differential to check for infection or anemia; measurement of electrolytes; and if other investigations are not conclusive, brain imaging (MRI or computerized tomography) for ventriculomegaly, atrophy, or intracranial bleed is suggested [115]. It is acknowledged that communicating hydrocephalus (due to defective cerebrospinal fluid reabsorption) is less common in patients with Sanfilippo syndrome than in other MPS disorders and can be difficult to distinguish from brain atrophy in MRI scans [116]. However, given that patients with GAG accumulation in MPS I and MPS II also have abnormal cerebrospinal fluid reabsorption [117, 118], it is reasonable to consider as a potential cause of pain and distress in patients with Sanfilippo syndrome and should be investigated. In the evaluation of increased intracranial pressure, evidence of papilledema may indicate elevated pressure but is not a reliable indicator of chronically elevated intracranial pressure; therefore, a normal fundus does not exclude the presence of intracranial pressure-related symptoms.

Standardized pain assessments appropriate for the patient’s cognitive level and/or caregiver proxy assessments should be included in regular follow-up visits for patients with Sanfilippo syndrome. For patients who have a limited ability to communicate, the revised Non-Communicating Children's Pain Checklist is recommended [119].

#### Special education, physical, occupational, speech, and complementary therapy interventions

Patients with Sanfilippo syndrome have unique needs and developmental trajectories that require careful and informed consideration throughout their period of care. Changes in neurocognition, speech, language, and motor skills may be subtle and not obvious from day to day or even month to month. Without careful and consistent monitoring of these outcomes by appropriately trained individuals, which interventions are benefiting a patient and whether alternative approaches need to be adapted are impossible to determine. In this regard, the consistent use of established measurement tools is recommended to track motor skills over time (eg the Peabody Developmental Motor Scales II, the Bayley-III motor domain and the Bruininks–Oseretsky test of motor proficiency, second edition) [74], with the selection of measures that are most appropriate for the patient [120].

Whereas for many children who do not have neurodegenerative disorders, goals for receiving supportive interventions are set based on an anticipated improvement in relevant skills, the natural course of Sanfilippo syndrome means that children beyond a certain point in their disease process will instead either lose or never acquire such skills. Therefore, supportive interventions should strive to maintain existing skill levels rather than require improvement in a patient’s abilities for them to qualify for continued access to such services. Similarly, therapeutic goals for rehabilitative therapies such as physical or speech therapy should focus on prolonging skills for as long as possible and improving quality of life and functional access to educational and social environments. Cognitive impairment and the progressive nature of Sanfilippo syndrome should not preclude an affected patient’s access to vision, hearing, behavioral, or any other support services; the patient should be recommended access to these services even after a decline in the relevant skills and abilities.

The provision of an appropriate high-quality, enriching educational environment with regular opportunities for peer engagement helps to ensure maximal developmental gains and skill maintenance [9, 12, 67, 71]. Consistent routines and structured schedules can have a positive influence on the behavior and quality of life of patients with Sanfilippo syndrome. As much as possible, these patients deserve stimulation and inclusion, even when processes of deterioration have begun. Providing an individual aide in the school setting is helpful and often necessary to maintain the safety of the patient and others in the classroom, as well as to maximize the child's attention, adequately reinforce their attempts to communicate, and support them during educational activities.

Speech regression can contribute to the distress and frustration experienced by the patient and their family, with dysfluencies and speech apraxia as early warning signs of regression in patients with Sanfilippo syndrome [11]. An array of augmentative and alternative communication (AAC) methods can be used to augment, complement, or replace speech for patients with complex communication needs [121]. Such methods range from basic tools (eg picture boards and single voice output buttons) to smart devices and dedicated AAC devices that integrate hardware and software to support a patient’s communication needs. The potential benefits of these approaches should be considered on a patient-by-patient basis, noting that patients with Sanfilippo syndrome may require longer and more intensive therapy to achieve success with these methods. The above approaches should be applied to patients with Sanfilippo syndrome as early as possible (ie during maximal cognitive capacity and even prior to loss of verbal speech). AAC methods should be initiated by a trained professional on a trial basis to determine suitability and feasibility, and then generalized for use in home and educational settings as quickly as possible. The type of AAC may need to be adjusted over time per the patient’s need, ability, and level of engagement. Behavioral therapies are helpful in conjunction to increase acceptance and positively reinforce the use of these communication tools.

Regular physical therapy may reduce physical discomfort and support some aspects of mobility in patients with Sanfilippo syndrome, which can have beneficial effects on inattention or other behaviors that may be driven by pain or frustration, as well as bone health, gastrointestinal motility, avoidance of pressure sores, and enabling them to maintain access to their environment. Thus, it is our opinion that physical therapy should be considered as early as possible and be performed regularly prior to, during, and beyond the decline of gross motor skills to maintain mobility and function and reduce development of contractures. The range of motion in the upper and lower extremities should be assessed at diagnosis, on the first visit to any new physical therapy provider, and at least every 6 months. Orthotic bracing may help balance, foot and ankle positioning, and to improve and maintain gait function and mobility for longer.

To help facilitate daily activities for patients with Sanfilippo syndrome, supportive equipment needs should be discussed, and appropriate prescriptions and referrals as needed made every 6 months. A forward-looking, proactive approach is warranted to ensure that required adaptive equipment can be obtained when needed, including a wheelchair or medical stroller, stander, bath seat, activity chair, safety beds, lifts, or specialized car seats. Similarly, adaptations to the home or school environment may be needed owing to the patients’ lack of safety sense and cognitive decline with preserved motor skills.

#### Growth

Although patients with Sanfilippo syndrome are generally of normal weight and height for their gestational age at birth, adults with Sanfilippo syndrome are generally of short stature [122, 123]. In a study of 182 patients with Sanfilippo syndrome in Germany, accelerated growth was observed in the first year of life, followed by decelerated growth from 4.5 to 5.0 years of age onwards. On reaching adulthood, these patients were shorter than expected based on the height of their respective parents [123]. Similarly, growth charts of patients with Sanfilippo syndrome in the Netherlands showed significantly slowed growth from 6 years of age onwards [122]. Disease-specific growth charts are important tools for tracking growth and recognizing deviation from normal and help physicians to counsel parents regarding growth expectations. Therefore, growth should be monitored and plotted on Sanfilippo syndrome-specific growth curves [123].

#### Puberty

The onset of puberty may be advanced in patients with Sanfilippo syndrome [124, 125]. If signs of early puberty are present, referral to a pediatric endocrinologist is warranted. The use of gonadotrophin-releasing hormone agonists is not contraindicated for patients with Sanfilippo syndrome and should be considered in consultation with the patient and their family.

#### Family support

Coming to terms with a diagnosis of Sanfilippo syndrome—a condition that many people may never have heard of—can be incredibly challenging for family members. Caregivers should be provided with counseling on the natural history of disease progression in the absence of a disease-modifying treatment, both at the time of diagnosis and throughout the disease course. Understanding what the expected symptoms of the disease are can help to ‘normalize’ the patient’s challenging behaviors, sleep, and other concerns during other times of high stress or changing symptom patterns [53, 126, 127]. However, this should not supplant the need to assess for treatable modifiers of symptoms that may improve the quality of life for the patient and their family.

Given that family members living with or caring for an individual with Sanfilippo syndrome will most likely experience significant psychological stress and social challenges, proactive intermittent assessment of caregivers’ anxiety, depression, and chronic traumatic stress with appropriate referral is warranted [126]. Patient advocacy groups provide a forum for peer-to-peer support and can facilitate the provision of services and financial aid grants available from the government and other community resources. Palliative care teams should be engaged, with regular monitoring of the service needs of the family and patient as these needs vary in intensity and type depending on the age and extent of disease progression.

## Discussion

While there are no approved therapies for patients with Sanfilippo syndrome, disease-specific therapies are being developed and a number of clinical trials have been attempted or are ongoing (eg intravenous and intrathecal enzyme replacement therapy, substrate reduction therapy, autologous stem cell-based lentiviral gene therapy, and adeno-associated viral vector gene therapy). In lieu of the promise provided by these novel therapies, this review aims to distill the best available guidance on how to recognize, diagnose, and care for patients with this devastating, progressive, and life-limiting disease based on the broad consensus of a multidisciplinary panel of expert clinicians from nine countries.

To our knowledge, this is the first example of a consensus guideline for the management of patients with Sanfilippo syndrome. Its development was led by a steering committee consisting of internationally renowned experts in Sanfilippo syndrome. The recommendations described here reflect the current understanding and experience of caring for patients with this condition. While guidelines support consistency in care, clinical judgment should be used to determine if deviation from the described schedule is appropriate based on the patient’s clinical history, extent of organ manifestations, variability of disease phenotype, and in collaboration with the family as to the potential burden of assessments.

It is acknowledged that a potential limitation of these guidelines is the fact that they do not fully encapsulate local variations in terminologies and different cultural systems of care, as some modifications to guideline statements were needed to ensure their applicability across the global healthcare landscape. Furthermore, given the large number of statements required to capture the available published evidence and experience of current clinical practice, not all issues and theories associated with the management of Sanfilippo syndrome could be covered within this review, although a full list of the recommendations that reached consensus are provided in Additional file 1: Table S2. Some of the statements put forward for consideration did not reach consensus owing to variations in local practice or lack of supporting evidence. For example, consensus was not reached on the appropriateness of routine monitoring of bone density using a dual-energy X-ray absorptiometry scan to assess for fracture risk in patients with prolonged functional immobility. Similarly, routine monitoring for retinal disease via electroretinogram and/or optical coherence in patients who do not have overt visual impairment remains a subject of debate. However, we do not consider that the guidance provided in this document is affected substantially by the omission of these statements. As is common in most rare diseases, some features of the disease are not as well represented in the literature as others. In such areas, there is an increased risk of bias from expert opinion based on clinicians’ individual clinical experience. We attempted to mitigate the impact of this potential bias by including a large number of clinicians with significant experience in Sanfilippo syndrome across a wide range of specialties and geographic locations so that their collective experience would offer a more comprehensive perspective.

Family and patient burden is an important consideration in medical-decision making. The extent of burden that is experienced or anticipated by each patient and family unit is unique. Further, benefits of monitoring procedures may not be immediately evident but rather may be appreciated at a later time point when disease symptoms evolve and the team then has a baseline from which to compare, allowing for better-informed clinical decisions. In this set of guidelines, we aim to respect the autonomy of patients and families by bringing consensus recommendations for proactive care together in one document, thus allowing them to make their own informed, individual decisions regarding benefit–risk–burden calculations in consultation with their care team.

As clinical experience of managing patients with Sanfilippo syndrome continues to grow, along with our understanding of the underlying disease pathways, the strategies described in this review will most likely require updates to reflect the closing of remaining knowledge gaps. The continued study of patients with Sanfilippo syndrome via observational studies, clinical registries, and preclinical studies is essential to ensure that progress continues to be made. Ultimately, the availability of the first disease-specific therapies for Sanfilippo syndrome will result in a major transformation of the clinical landscape and prospects for the patients and families affected by this disorder. The guidance contained here should therefore be reviewed and updated regularly by a panel of appropriately qualified experts.

## Conclusions

Sanfilippo syndrome is a complex neurodegenerative disease that, until now, had no published standard global clinical care guideline. This document, created through collaboration between Cure Sanfilippo Foundation (USA) and Sanfilippo Children’s Foundation (Australia), distills 178 guideline statements into an easily digestible document that provides evidence-based, expert-led recommendations. This review is intended to help the provision of consistent care to the patients and families affected by Sanfilippo syndrome, as well as facilitating interventions to improve their quality of life.

## Supplementary Information

**Additional file 1**. Supplemental methods and results.

## Data Availability

All data generated or analyzed during this study are included in this published article and its supplementary information files.
