# Supplementary material for: Sanfilippo syndrome: consensus guidelines for clinical care
Source: Orphanet J Rare Dis. 2022 Oct 27;17:391. doi: 10.1186/s13023-022-02484-6 (PMC9612603; doi:10.1186/s13023-022-02484-6)
Supplement: Supplementary file 1 — Additional file 1. Supplemental methods and results. [file 13023_2022_2484_MOESM1_ESM.docx]

**SUPPLEMENTAL MATERIAL**

**Sanfilippo syndrome: consensus guidelines for clinical care Literature search terms used**

(“MPSIII” OR “mucopolysaccharidosis type III” OR “Sanfilippo syndrome” OR “Sanfilippo disease”) AND (“management” OR “care” OR “treatment” OR “therapy” or “natural history” or “behavior” or "behaviour" OR “sleep” or “orthopaedic” or “hearing” OR "anaesthetic" OR "pulmonary" OR "gastrointestinal" OR "occupational therapy" OR "physiotherapy" OR "physical therapy" OR "dentistry" OR "teeth" OR "dentist" OR "orofacial" or "oral" OR "palliative" OR "ophthalmology" OR "vision" OR "cardiac" OR "nutrition" OR "respiratory" OR "pain" OR "neurologic" OR "seizure" OR "spasticity" OR "swallowing" OR "autism" OR "psychology" OR "otolaryngology" OR "audiology" OR "otitis" OR "behavioral therapy" OR "applied behavioral analysis" OR "hydrotherapy" OR "hippotherapy" OR "speech therapy" OR "heart" OR "cardiovascular" OR "MRI" OR "imaging" OR "puberty" OR "stools" OR "diet" OR "bone density")

**Table S1** Guideline development group members

| **Name** | **Specialty** | **Institution** | **Country** |
| --- | --- | --- | --- |
| Mehmet Umut Akyol | Otorhinolaryngology | Hacettepe University, Ankara | Turkey |
| Reshma Amin | Pediatric Respirology and Sleep Medicine | The Hospital for Sick Children (SickKids), Toronto, ON | Canada |
| Jane Ashworth | Ophthalmology | Manchester Royal Eye Hospital, Manchester | UK |
| Kelly Bossola | Physical therapy | UPMC Children’s Hospital of Pittsburgh, Pittsburgh, PA | USA |
| Elizabeth Braunlin | Pediatric cardiology | University of Minnesota, Minneapolis, MN | USA |
| Sandra Breyer | Pediatric orthopedic surgeon/surgery | University Medical Center Hamburg-Eppendorf, Hamburg | Germany |
| Ana Carolina Brusius-Facchin | Molecular Biology, Medical genetics | Hospital de Clinicas de Porto Alegre, Porto Alegre, RS | Brazil |
| Maira Burin | Biochemistry, Medical genetics | Hospital de Clínicas de Porto Alegre, Porto Alegre, RS | Brazil |
| Marco Corridore | Anesthesiology and pain medicine | Nationwide Children's Hospital, Columbus, OH | USA |
| Megan Donnell | Patient advocate | Sanfilippo Children’s Foundation, Freshwater, NSW | Australia |
| Elise Drake | Patient advocate | Cure Sanfilippo Foundation, Columbia, SC | USA |
| Carolyn Ellaway | Clinical and metabolic genetics | Sydney Children's Hospital Network, Sydney, NSW | Australia |
| Kristina Elvidge | Patient advocate | Sanfilippo Children’s Foundation, Freshwater, NSW | Australia |
| Maria Fuller | Biochemical genetics | SA Pathology at Women’s and Children’s Hospital, Adelaide, SA | Australia |
| Roberto Giugliani | Medical genetics | HCPA, UFRGS, DASA, Casa dos Raros, Porto Alegre, RS | Brazil |
| Livia Goldraich | Cardiology | Hospital de Clínicas de Porto Alegre, Porto Alegre, RS | Brazil |
| Lynn Golightly | Speech-Language Pathologist, Dysphagia Therapist | UPMC Children’s Hospital of Pittsburgh, Pittsburgh, PA | USA |
| Daniel Guillaume | Neurosurgery | University of Minnesota, Minneapolis | USA |
| Dougal J Hare | Psychology | University of Manchester and Helios-UK, | UK |
| Rebecca Howarth | Speech, Language, Dysphagia Therapy | Royal Manchester Children’s Hospital, Manchester | UK |
| Simon A Jones | Pediatric genetics and metabolism | Royal Manchester Children’s Hospital, Manchester | UK |
| Till Koehne | Orthodontics | University Medical Center Leipzig, Leipzig | Germany |
| Diane Ruschel Marinho | Ophthalmology | Hospital de Clínicas de Porto Alegre (UFRGS), Porto Alegre | Brazil |
| Eleanor Mc Govern | Paediatric Dentistry | Children’s Health Ireland at Temple Street, Dublin | Ireland |
| Lisa Melton | Patient advocate | Sanfilippo Children’s Foundation, Freshwater, NSW | Australia |
| Joseph Muenzer | Pediatric genetics and metabolism | University of North Carolina, Chapel Hill, NC | USA |
| Nicole Muschol | Pediatric genetics and metabolism | University Medical Center Hamburg-Eppendorf, Hamburg | Germany |
| Cara O’Neill | Pediatrics, Patient advocate | Cure Sanfilippo Foundation, Columbia, SC | USA |
| Mark Pertini | Neuropsychology | Women’s and Children’s Hospital, Adelaide, SA | Australia |
| Lynda Polgreen | Pediatric endocrinology | Lundquist Institute at Harbor-UCLA Medical Center, Torrance, CA | USA |
| Elsa Shapiro | Pediatric neuropsychology | University of Minnesota, Minneapolis, MN and Shapiro Neuropsychology Consulting, Portland, OR | USA |
| Nicholas Smith | Pediatric neurology | Women’s and Children’s Hospital, Adelaide, SA | Australia |
| Martha Solano | Pediatric neurology | Fundación Cardioinfantil - Instituto de Cardiología, Bogotá | Colombia |
| Carolina Fischinger Moura de Souza | Medical genetics | Hospital de Clínicas de Porto Alegre, Porto Alegre, RS | Brazil |
| Michel Tchan | Clinical and metabolic genetics | Westmead Hospital, Sydney, NSW | Australia |
| Mohammed Usman | Paediatric Psychiatry | Women’s and Children’s Hospital, Adelaide, SA | Australia |
| Robert Walker | Anesthesia | Royal Manchester Children's Hospital, Manchester | UK |
| Rachel Ward | Pediatric Audiology | Manchester Royal Infirmary, Audiology (Hearing & Balance) Centre, Manchester | UK |
| Chet Whitley | Pediatric genetics and metabolism | University of Minnesota, Minneapolis, MN | USA |
| Michelle Wood | Physiotherapy | Great Ormond Street Hospital, London | UK |
| Timothy Wood | Biochemical and molecular genetics | University of Colorado/Children's Hospital of Colorado, Aurora Colorado | USA |

UCLA, University of California Los Angeles; UPMC, University of Pittsburgh Medical Center

**Table S2** Recommendations (core and supplemental) and level of consensus

Consensus recommendations have been divided into 156 core statements that tackle the most pressing needs faced by patients with Sanfilippo syndrome, and 22 supplemental statements that address some of the less common aspects of diagnosing and managing the disease, or areas for which further evidence is still needed. Consensus is defined as 75% or more responses of ‘Strongly Agree’ or ‘Agree’, excluding ‘not my area of expertise’.

**A. Diagnosis**

| **Core/ supplemental** | **Recommendation** | **Agree or strongly agree** |
| --- | --- | --- |
| Core | In the case where a family seeks prenatal diagnosis, testing modalities should include molecular genetic testing and enzyme activity testing, being strongly indicated to use both methods whenever possible. For cases where there is an older sibling with confirmed MPS III diagnosis by molecular testing and there are two known mutations, prenatal diagnosis may be made with molecular testing alone | 87.0%  (40/46) |
| Core | Newborn screening is critical to pre-symptomatic diagnosis, enabling timely intervention with optimal outcomes when specific therapies are approved, and its adoption should become standard of care (Note: Currently, there are ongoing newborn screening pilot studies for MPS IIIA and MPS IIIB) | 80.0%  (40/50) |
| Core | Recommend high index of suspicion and increased awareness efforts to reduce time to diagnosis (Note: Early diagnosis allows for specific supportive interventions to improve quality of life, opportunities to participate in clinical trials and/or receive treatments as they emerge and timely genetic counseling of affected families) | 94.3%  (50/53) |
| Core | For patients with speech delay and/or non-specific developmental delays, an underlying diagnosis of Sanfilippo syndrome should be considered | 89.5%  (51/57) |
| Core | Sanfilippo syndrome is to be considered in the differential diagnosis for behavioral/psychiatric findings such as: hyperactivity, ADHD, autistic behaviors, sleep disturbance, and intellectual disability | 94.8%  (55/58) |
| Core | Sanfilippo syndrome is to be considered in the differential diagnosis of physical findings such as: hearing loss, hepatosplenomegaly, macrocephaly, coarse facial features, umbilical hernia, recurrent ENT infections, diarrhea or chronic loose stools, retinitis pigmentosa, cardiomyopathy, valvular heart disease, persistent tachypnea in the neonate, and/or sleep apnea | 94.5%  (52/55) |
| Core | Patients requiring more than one set of ear grommets for persistent middle ear effusion or infection, and sensorineural hearing loss and/or need for earlier than usual adenotonsillectomy should be evaluated for MPS III | 75.6%  (34/45) |
| Core | Sanfilippo syndrome should be considered in patients of all ages, not just children since slower progressing forms of the disease are noted. Investigation for MPS III is warranted in adults who show signs of early onset dementia, vision loss with retinitis pigmentosa, and/or adult-onset cardiomyopathy | 93.9%  (46/49) |
| Core | Behavioral, cognitive, and physical findings in MPS III occur across a spectrum from severe early onset/rapidly progressing to slower progressing forms with survival into adulthood | 94.5%  (52/55) |
| Core | When there is suspicion for Sanfilippo syndrome (MPS III) patients should receive prompt referral to a genetic or metabolic center | 96.6%  (56/58) |
| Core | Screening/diagnostic tests should be initiated by the primary care provider so as not to delay time to diagnosis, in conjunction with referral to genetic/metabolic specialist | 87.3%  (48/55) |
| Supplemental | Neuroimaging and radiology findings can be strongly indicative of MPS III however, are not pathognomonic and rarely specific in isolation, necessitating disease confirmation via dedicated biochemical and molecular assays | 93.5%  (43/46) |
| Supplemental | Radiology and neuroimaging can often appear normal in the early stages of the disease, as such, an unremarkable imaging result does not exclude an MPS III diagnosis | 97.8%  (45/46) |
| Core | Analysis of urine glycosaminoglycans (GAGs) using quantitative and qualitative analysis of urine glycosaminoglycans in a first morning void sample are accepted biochemical diagnostic tests. A random timed sample is also acceptable, and a sterile sample is not required.   - The recommended quantitative GAG assay is the DMB (dimethylmethylene blue) (or the assay of uronic acid, in some countries) - The use of age-related reference ranges is highly recommended due to the natural decrease in GAG levels with age, both in patients and normal subjects - The recommended qualitative GAG assay is GAG electrophoresis iv) Urine GAG tests are screening tests and cannot rule out MPS III due to significant rate of false-negatives, therefore in cases of high clinical suspicion with a negative urine GAG screen, follow-up with enzyme analysis or genetic testing is recommended | 93.0%  (40/43) |
| Supplemental | The qualitative identification of heparan sulfate, by electrophoresis or thin-layer chromatography, may play a role in MPS III screening, especially in older patients who may have normal or near normal total urinary GAGs | 85.3%  (29/34) |
| Core | Enzyme analysis of all four enzymes implicated in MPS III via a blood or fibroblast sample is the recommended gold standard for confirmation of diagnosis | 89.7%  (35/39) |
| Core | The assay of all 4 MPS III enzymes in blood or fibroblasts should be performed in cases with increased GAGs and/or presence of heparan sulfate | 89.5%  (34/38) |
| Core | All enzymes can be assayed simultaneously or can be ordered in a sequence according to the relative frequency of the MPS III types in the region, or for logistical reasons | 85.3%  (29/34) |
| Core | Enzyme analysis can be considered as a first-line test, particularly in situations where it is difficult to obtain a suitable urine sample and/or to ship it in adequate conditions | 82.1%  (32/39) |
| Core | If the enzyme for MPS IIIA or MPS IIID are deficient (both sulfatases), at least one other sulfatase should be assayed to rule out additional sulfatase deficiencies | 94.6%  (35/37) |
| Supplemental | In the case of MPS IIIC, hexosaminidase should be included in enzyme testing since impaired beta-hexosaminidase activity can produce a false-positive result for MPS IIIC | 89.3%  (25/28) |
| Core | A positive result in dried blood spot (DBS) samples should be confirmed by an enzyme assay in leucocytes or fibroblasts, and/or by molecular genetic analyses | 89.7%  (35/39) |
| Core | Molecular genetic testing should be offered to all patients because:   - It enables cascade molecular screening of undiagnosed siblings or extended family members, and family members who are carriers, enabling appropriate genetic counseling for future family planning - It may inform clinical expectations of disease progression, according to the pathogenicity of the mutation and knowledge about genotype–phenotype correlation - Molecular testing results may impact the patient’s eligibility for clinical trials and future therapeutic treatments | 95.5%  (42/44) |
| Core | Cases with a primary molecular genetic diagnosis should have a confirmatory enzyme activity test and/or an analysis of urinary GAGs to confirm the pathogenicity of the mutations when a variant of unknown significance is found | 97.6%  (40/41) |
| Supplemental | Molecular genetic testing of both biological parents and the affected patient is preferred to only testing the affected patient, whenever possible | 90.5%  (38/42) |
| Core | Confirmation of diagnosis requires positive indicators of disease on two modes of testing: (ie urine GAG + enzyme activity; urine GAG + molecular testing; enzyme activity + molecular testing) | 90.0%  (36/40) |
| Core | In the presence of homozygosity for a known pathogenic MPS III mutation or heterozygosity for two known pathogenic mutations, a diagnosis can be made with reasonable confidence in a patient expressing a phenotype consistent with MPS III | 89.7%  (35/39) |

**B. Neurology**

| **Core/ supplemental** | **Recommendation** | **Agree or strongly agree** |
| --- | --- | --- |
| Core | Regular neurologic assessments are recommended at baseline then every 6–12 months, and more frequently if clinically indicated. Monitor gross motor, fine motor, tone, sleep disturbances, seizure activity, disorders of movement and behavioral changes | 96.3%  (52/54) |
| Core | Walking/gait should be assessed at baseline then every 6–12 months and as needed. Monitor for functional impairments, interval therapy needs, medical equipment needs, bracing and other referrals | 94.1%  (48/51) |
| Core | Children may require more time to initiate or complete a task due to the development of motor apraxia and challenges with motor planning which occur in MPS III disease progression. This should be accommodated for in clinical exams, formal testing, and during educational and therapeutic activities | 98.1%  (53/54) |
| Core | High-resolution MRI of the brain should be performed at baseline and as clinically indicated. [Note: Neurodegeneration in MPS III patients can be represented by decreases in cortical volume and increases in ventricular volume on MRI over time] | 76.1%  (35/46) |
| Core | Triggers for ordering an MRI of the brain may include extreme behavioral changes, unexplained pain or distress, suspicion of headaches, suspicion of elevated intracranial pressure, sudden neurological or functional declines, etc | 95.7%  (45/47) |
| Supplemental | If diagnosed with MPS III in infancy, monitor for craniosynostosis | 76.2%  (30/39) |
| Core | Recommend that clinicians monitor for and have a high index of suspicion for the development of movement disorders such as dystonia, ataxia, and dyskinesias (including tics, myoclonus, and choreoathetosis) | 87.5%  (42/48) |
| Core | Recommend that clinicians have a high index of suspicion in monitoring for epileptic activity (convulsive and non-convulsive) in patients with MPS III. Seizures can be subtle and may be difficult to recognize in patients who have cognitive deficits and significant baseline behavioral differences | 100%  (51/51) |
| Core | Both convulsive and non-convulsive epilepsy should be adequately treated according to the patient's individual needs and medication history | 97.9%  (46/47) |
| Core | Preference should be given to anti-epileptic drugs with fewer drug-drug interactions and which do not require therapeutic drug level monitoring | 95.3%  (41/43) |

**C. Considerations for neurobehavior, psychological and psychiatric care**

| **Core/ supplemental** | **Recommendation** | **Agree or strongly agree** |
| --- | --- | --- |
| Core | Targeted behavioral/psychiatric medication prescribing should be accompanied by an evaluation of contraindicated risks, which is especially important given that MPS III is a multisystem disease and the impact of psychotropic medication on cardiac, hepatic and renal systems needs to be taken into account | 97.9%  (46/47) |
| Core | Recommend identifying and targeting psychiatric symptom clusters in the context of the developmental age equivalent profile of each child. Clusters include sleep, ADHD behaviors, social communication difficulties, speech and language difficulties, sensory difficulties and anxiety | 93.3%  (42/45) |
| Core | Regular monitoring of neurocognitive function is recommended to help families identify loss of skills and to support discussions focused on adjustment to disease progression, educational needs and supports in the later phases of the disease | 90.2%  (46/51) |
| Core | ABA (applied behavioral analysis) therapy, where available and tailored to the individual child, should be supported in order to enhance communication skills, maintain motor abilities, and reduce unsafe behaviors as it has been found to be beneficial for some children with Sanfilippo syndrome | 90.7%  (39/43) |
| Core | When evaluating and monitoring adaptive behavior skills, it is recommended to use the Vineland Adaptive Behavior Scale as at least one of the measures | 83.7%  (36/43) |
| Core | When considering behavior-modifying medications, careful consideration of any physical problem such as pain, musculoskeletal problems, gastrointestinal disturbances, seizures, dental problems, communication challenges, etc is necessary to formulating a proper treatment strategy | 100%  (50/50) |
| Core | Use of stimulant medications, mood stabilizers, antipsychotics and antianxiety drugs may be considered on a case-by-case basis and with short-term trials after review of potential risks and benefits with the patient’s family | 95.7%  (45/47) |
| Core | Recommend developmental testing be performed in an environment familiar to the child by a tester who has established rapport with the patient and has familiarized themselves with the behavioral characteristics of MPS III prior to testing | 88.2%  (45/51) |
| Core | Behavioral symptoms require a holistic approach of understanding the behavior in context of the cognitive skill level, creating a safe home and school environment for the child, providing routine and structure in addition to any pharmacologic approaches | 100%  (50/50) |

**D. Respiratory management**

| **Core/ supplemental** | **Recommendation** | **Agree or strongly agree** |
| --- | --- | --- |
| Core | Recommend high index of suspicion for diagnosis of pneumonia and consideration of early diagnostic radiology since patients with MPS III do not always show typical signs of illness until infection becomes more overwhelming | 97.7%  (43/44) |
| Core | Recommend early treatment of pneumonia/suspected lung infection with antibiotics due to potential for rapid decompensation and difficulty monitoring clinical wellness in children with MPS III | 97.5%  (39/40) |
| Core | Routine childhood vaccinations should be recommended as well as annual flu shot and the 23-valent pneumococcal vaccine (Pneumovax 23) | 95.3%  (41/43) |
| Core | All individuals with MPS III should have baseline respiratory and sleep evaluation (note, if respiratory medicine and sleep medicine are not combined, separate respiratory medicine and sleep referrals are recommended) | 93.0%  (40/43) |
| Core | Baseline respiratory system focused history should include the following: sleep hygiene, quality and duration, symptoms of sleep disordered breathing, history of respiratory symptoms (eg chronic cough), history of pneumonia, history of oral secretions, history of difficulty feeding, history of gastroesophageal disease and history of nasal secretions/ congestion should be reviewed | 95.2%  (40/42) |
| Core | Baseline physical examination should include vital signs (including respiratory rate, heart rate, height, weight, oxygen saturations and non-invasive carbon dioxide measure if available) and routine respiratory examination | 100%  (44/44) |
| Core | Diagnosis and management of sleep apnea should be under the guidance of a respiratory physician. This may require the introduction of CPAP or other interventions | 82.2%  (37/45) |
| Core | Ongoing respiratory and sleep medicine follow-up is recommended. The frequency of which will depend on the severity of the respiratory disease and sleep disordered breathing | 90.9%  (40/44) |
| Core | Excessive oral secretions may be managed by manual suction and/or medications such as atropine or glycopyrrolate | 89.4%  (42/47) |

**E. Sleep**

| **Core/ supplemental** | **Recommendation** | **Agree or strongly agree** |
| --- | --- | --- |
| Core | In patients with sleep disturbance, the medical workup should include consideration of the presence of disordered movement or seizure activity, iron deficiency in the setting of restless legs, pain or intercurrent illness, esophageal reflux, dental disease and disordered breathing during sleep | 97.8%  (44/45) |
| Core | Circadian rhythm disruption is a well-accepted complication of Sanfilippo syndrome that may be partly addressed by melatonin supplementation | 97.6%  (41/42) |
| Core | If melatonin is started, it is recommended to begin at low doses of 0.5 mg and titrate to effect up to a maximum dose of 12 mg | 85.0%  (34/40) |
| Core | Sleep disturbance in Sanfilippo syndrome should be addressed with a multimodal approach to include sleep hygiene counseling, implementing behavioral strategies, addressing safety of the environment, treating circadian rhythm disturbance and other comorbid medical factors | 100%  (46/46) |
| Core | Use of sleep diaries is encouraged for monitoring changes, evolution of sleep disturbance and response to interventions | 84.4%  (38/45) |

**F. Anesthesia and peri-operative care**

| **Core/ supplemental** | **Recommendation** | **Agree or strongly agree** |
| --- | --- | --- |
| Core | Sedation/anesthesia events should always be conducted in the hospital setting with experienced anesthesia personnel available and ready to manage complex airway emergencies | 94.3%  (50/53) |
| Core | All attempts should be made to coordinate procedures and diagnostics which will require sedation with the multidisciplinary team such that the number of anesthesia events is minimized | 98.1%  (54/55) |
| Core | Observe patient-centered accommodations for those with behavioral challenges and neuro-disability to include:   - Parent/caregiver access to patient through anesthesia induction and upon emergence/recovery - Provision for low-stimulus environment with ability to secure/close door to reduce risk of child escaping - Use of distraction techniques and items - Consider safety concerns with regards to impulsivity, hyperactivity and flight risk - Provision for additional supervisory staff as appropriate to meet patient’s individual needs | 96.4%  (53/55) |
| Core | Sedation/general anesthesia with a native airway (without pharyngeal or laryngeal intubation) may be considered for patients with MPS III, though the use of standard airway maneuvers and adjuncts (ie chin lift, shoulder roll, CPAP, oral/nasal airways) may be needed | 86.1%  (31/36) |
| Core | Laryngeal Mask airway is a good alternative to tracheal intubation for many MPS III patients in whom a native airway is not feasible | 85.3%  (29/34) |
| Core | When preparing for anesthesia in the MPS III patient, providers should always be prepared for the potential of difficult laryngoscopy and intubation | 93.0%  (40/43) |
| Core | Unless contraindicated, chronic medications should be given on the day of anesthesia within the confines of fasting guidelines, particularly anticonvulsants and neurobehavioral medications | 97.9%  (46/47) |
| Core | Pre-operative anesthetic review and airway assessment should be conducted prior to the day of scheduled procedure to allow time to have any necessary equipment and staff available for the sedation event | 95.8  (46/48) |
| Supplemental | Cardiology review: in the absence of specific clinical concern, a pre-op/pre-anesthesia echocardiogram and electrocardiogram are recommended if no prior studies have been performed within the preceding 24 months (Echo) or 12 months (ECG) | 88.1%  (37/42) |
| Supplemental | Pulmonary/respiratory: consider patient’s history of chronic respiratory disease, asthma, or episodes of post-sedation respiratory distress. (Note: Glossal hypertrophy is a known complication in some patients with MPS III [39% in one natural history study] and should be considered in the airway assessment) | 95.6%  (43/45) |
| Supplemental | Heme review: for invasive procedures, pre-operative complete blood count and coagulation profile (PT, PTT) is recommended if not done in the preceding month | 76.6%  (36/47) |
| Supplemental | Neurologic review: note patient’s baseline neurologic status to include presence of autonomic instability, seizures, spasticity, movement disorder, neurobehavioral symptoms and associated medications | 98.0%  (50/51) |

**G. ENT/audiology considerations**

| **Core/ supplemental** | **Recommendation** | **Agree or strongly agree** |
| --- | --- | --- |
| Core | Otolaryngologists and audiologists should consider the diagnosis of MPS III in children presenting with recurrent otitis, chronic nasal congestion, and any degree of hearing loss | 88.4%  (38/43) |
| Core | Early and aggressive management of hearing impairment and ear effusion should be performed to optimize language development during critical developmental windows and reduce impact on behavioral problems | 98.0%  (50/51) |
| Core | ENT examination and audiologic testing should be done at baseline just after diagnosis | 96.2%  (51/53) |
| Core | Audiology evaluation should include assessments of both air and bone conduction | 93.5%  (43/46) |
| Core | Where hearing assessment is needed and behavioral testing is not possible, ABR testing under sedation or general anesthesia should be considered | 87.0%  (40/46) |
| Core | ENT and audiology follow-up of patients should occur at least every 12 months and more frequently if there are recurrent episodes of otitis or suspected changes in hearing | 93.5%  (43/46) |
| Core | When there is identified hearing loss or otitis media with effusion, follow-up should be more frequent and at least every 6 months, based on the individual child | 93.5%  (43/46) |
| Supplemental | If undergoing anesthesia, consider closer evaluation of the airway with fiberoptic scope to monitor progression of airway obstruction | 85.4%  (35/41) |
| Core | If the patient has signs and symptoms of obstructive sleep apnea along with adenoid and/or tonsillar hypertrophy, removal of adenoids and/or tonsils should be performed without delay | 84.6%  (33/39) |
| Core | Continuous positive airways pressure (CPAP) therapy should be considered for patients who display the presence of obstructive sleep apnea (OSA) which persists after adenoidectomy and/or tonsillectomy regardless of behavioral challenges | 86.0%  (37/43) |
| Core | Consultation with anesthesiology and multidisciplinary team should be done before (ENT) surgery | 96.0%  (48/50) |
| Core | The hearing needs of the child should always be clearly documented in the child’s records and care plans with accompanying advice on communication and hearing supports, particularly in the educational setting | 100%  (55/55) |
| Supplemental | Any surgery, especially airway procedures, should be done as soon as possible after the diagnosis of the patient, before morphologic changes due to GAG accumulation in and around the airway take place, to decrease the complication rate, morbidity and mortality | 78.0%  (32/41) |
| Core | If conductive type of hearing loss is detected due to effusion in the ear (lasting more than 2 months bilaterally or 4 months unilaterally), grommets (ventilation tubes) should be inserted without delay to maximize hearing and reduce symptoms | 89.5%  (34/38) |
| Supplemental | The increased likelihood of return of the otitis media with effusion (OME) once the grommets have fallen out should be taken into account when considering the use of longer-term ventilation tubes | 94.7%  (36/38) |
| Core | ‘Behind the ear’ (BTE) hearing aids should be considered for patients with hearing loss | 87.5%  (35/40) |
| Core | Behavior symptoms of the child should not be used as an excuse to not trial hearing aids, especially in the educational setting | 92.6%  (50/54) |
| Core | Decisions on use of hearing devices should be made in close collaboration with the family | 98.1%  (53/54) |
| Supplemental | Bone conduction hearing devices may be considered for patients who do not tolerate BTE hearing aids and have a significant component of conductive hearing loss. Bone conduction devices will not assist with sensorineural hearing loss | 81.8%  (27/33) |
| Core | ENT surgery remains a fundamental therapeutic procedure for reducing the frequency and severity of ear infections and for relieving the symptoms of upper airway obstruction, even if these interventions are not definitive | 87.5%  (35/40) |

**H. Ophthalmology**

| **Core/ supplemental** | **Recommendation** | **Agree or strongly agree** |
| --- | --- | --- |
| Core | Recommend full ophthalmologic evaluation by pediatric ophthalmologist at time of diagnosis and yearly or sooner if clinically indicated | 90.7%  (39/43) |
| Core | Ophthalmologic assessment should include assessment of vision in both eyes, orthoptic assessment, refraction, examination of anterior and posterior segment of the eye to include examination of the cornea, retina and optic nerve; and measurement of intraocular pressure (IOP) | 94.4%  (34/36) |
| Core | If retinopathy is clinical suspected (due to symptoms of nyctalopia, visual field loss or reduction in vision, or signs of pigmentary retinal change), then electroretinogram may be helpful in confirming the diagnosis | 93.1%  (27/29) |
| Core | Some patients with significant behavioral challenges will require examination under anesthesia in which case risk/benefit must be weighed | 95.5%  (42/44) |
| Core | Patients with MPS III and retinal/visual impairment should be provided access to low vision supports and services in the home, community, and educational settings. Vision impairments and supports should be included as part of the individualized educational plan (IEP) | 95.2%  (40/42) |

**I. Dental**

| **Core/ supplemental** | **Recommendation** | **Agree or strongly agree** |
| --- | --- | --- |
| Core | Since patients with MPS disorders are at high risk for dental disease, basic good oral hygiene is recommended with twice daily brushing and avoidance of regular sugary drinks | 98.0%  (49/50) |
| Core | In patients who have challenges clearing food from the oral cavity or who take daily sweetened liquid medications, offer water or wiping of the teeth after meals and snacks | 97.8%  (44/45) |
| Core | Dental sealants are recommended for MPS III patients to prevent and/or arrest dental caries in primary and/or permanent molars | 90.0%  (27/30) |
| Core | Dental sealants should be monitored for integrity at each dental visit and restored as indicted | 88.2%  (30/34) |
| Supplemental | Monitor for development of an open bite in children with MPS III with prolonged pacifier use | 84.6%  (33/39) |
| Supplemental | Alternative oral sensory chewing devices, specifically designed for this need, are preferred alternatives to pacifier use | 92.7%  (38/41) |
| Core | As brushing the teeth can be challenging in patients who dislike or do not understand this task, supports such as use of 3-sided toothbrush, bite block, and distraction techniques may be helpful | 95.2%  (40/42) |
| Core | Regular dental counseling, preventive applications and dental treatment must be included in the multidisciplinary team approach of MPS III patients | 100%  (49/49) |
| Supplemental | Gingivitis may be treated with chlorhexidine application to gums with an oral sponge | 76.7%  (23/30) |
| Core | If sedation is required for dental procedures, this should take place in a tertiary care facility with experienced anesthesia staff | 91.5%  (43/47) |
| Core | Oral health problems should be ruled out in the setting of behavioral changes, agitation, distress, change in sleep patterns, change in eating habits, or a change in oral sensory behaviors | 98.0%  (50/51) |

**J. Nutritional and gastrointestinal management**

| **Core/ supplemental** | **Recommendation** | **Agree or strongly agree** |
| --- | --- | --- |
| Core | Recommend monitoring patients for gastroesophageal reflux and considering trial of anti-reflux medication and/or diet modification in the setting of increased behavioral distress, increased sleep disturbance, and/or other clinical signs are present | 97.7%  (42/43) |
| Core | Stooling issues can be a source of discomfort and distress for patients that may manifest through an increase in behavioral disturbances, heightened sleep disturbance or other alternative expressions of pain | 97.9%  (47/48) |
| Core | Non-infectious loose stools are common in patients with MPS III. This should be noted in the care plan, particularly for all care providers in the educational and therapeutic settings and should not be a cause for exclusion from those activities | 88.6%  (39/44) |
| Core | Constipation can be a source of discomfort and behavioral disturbances, particularly as the patient loses mobility and experiences more neurologic impairment. Therapeutic maintenance regimens should aim for consistent and adequate elimination to maintain patient comfort and health | 97.9%  (46/47) |
| Core | Elevations in liver enzymes and hepatomegaly are common findings in MPS III but typically do not  require intervention   - ALT levels in MPS III A&B may be seen up to 3.5-times upper limit of normal - AST levels in MPS III A&B may be seen up to 1.5-times upper limit of normal | 91.7%  (33/36) |
| Core | Hernias of the umbilicus and inguinal area are often found in patients with MPS III but do not routinely require intervention. Observation only and monitoring on routine exam is recommended unless they become large or symptomatic | 89.7%  (35/39) |
| Core | Dietician referral is recommended for patients who have significantly self-limited diet, those experiencing weight loss or poor growth, sensory needs limiting proper nutrition, decline of oromotor skills impairing normal caloric intake in a reasonable time frame. Often referral should be made in conjunction with referral/consultation of a Speech Therapy Feeding specialist | 95.9%  (47/49) |
| Core | In cases of inadequate nutrition via oral feeding or presence of significant risk of aspiration or history of aspiration pneumonia, placement of an enteral feeding tube should be considered jointly with the family | 93.8%  (45/48) |
| Supplemental | Monitoring and restoration of micronutrient deficiencies is recommended to support metabolic functions | 90.2%  (37/41) |

**K. Cardiology**

| **Core/ supplemental** | **Recommendation** | **Agree or strongly agree** |
| --- | --- | --- |
| Core | All individuals with MPS III should have baseline cardiac evaluation at diagnosis to include a physical exam, vital signs, echocardiogram, and electrocardiogram | 96.0%  (48/50) |
| Core | Parents/caregivers should be encouraged to assist in strategies for calming and comforting the patient to complete the necessary tests and exam. If necessary, tests may be done under anesthesia when coordinated with other procedures | 94.5%  (52/55) |
| Core | Echocardiogram is recommended every 24 months if no abnormalities are noted at initial echocardiogram. If abnormalities are noted on initial or subsequent echocardiograms, frequency should increase to every 12 months. More frequent echocardiograms are to be performed as indicated based on the individual child's findings | 86.0%  (37/43) |
| Core | 12-lead electrocardiogram and rhythm strip is recommended every 12 months and as needed due to difficulty assessing symptoms in patients with MPS III (Note: storage of heparan sulfate in the cardiac conduction system may trigger AV block) | 81.0%  (34/42) |
| Core | If electrocardiogram is abnormal, a Holter monitor should be placed for 24-48 hours for a more thorough evaluation | 80.5%  (33/31) |

**L. Management of orthopedic complications**

| **Core/ supplemental** | **Recommendation** | **Agree or strongly agree** |
| --- | --- | --- |
| Core | Recommend pediatric orthopedic referral for baseline exam, scoliosis series X-rays and bilateral hip X-rays at the time of diagnosis and every 1–2 years from age 7 onwards or sooner, if clinically indicated. Patients with significantly attenuated disease phenotypes may need less frequent monitoring | 77.3%  (34/44) |
| Core | After initial baseline full spine films, routine monitoring for cervical spine instability is not recommended unless there is clinical suspicion | 90.2%  (37/41) |
| Core | Monitor for other orthopedic findings at routine yearly visits: trigger finger, genu valgus deformity, femoral anteversion and tibial torsion that do not appear to improve or if are worsening with age | 90.7%  (39/43) |
| Core | Secondary skeletal involvement in patients with MPS III may represent a considerable cause of morbidity (ie osteonecrosis of femoral head) and intervention should be considered on a case-by-case basis | 93.2%  (41/44) |
| Supplemental | MPS III patients with progressed disease features and significant mobility limitations, should have vitamin D metabolism assessed and treated as indicated to reduce fracture risk | 92.9%  (39/42) |
| Supplemental | Recommended *pes equinus* (toe-walking) management includes stretching and:   - Night-time orthoses, but if not tolerated then - Daytime orthoses, and if not effective or tolerated - Consider surgical lengthening or botox to improve mobility and reduce risk of falls | 87.5%  (35/40) |
| Supplemental | Orthopedic management of painful subluxation due to hip dysplasia in MPS III may be considered on a case-by-case basis | 100%  (42/42) |
| Core | Because pain may be difficult to assess and localize in MPS III patients with cognitive impairment and behavioral disturbances, radiographic studies of the hips should be considered in the evaluation of otherwise unexplained signs of discomfort or pain | 88.6%  (39/44) |

**M. Management of pain and distress**

| **Core/ supplemental** | **Recommendation** | **Agree or strongly agree** |
| --- | --- | --- |
| Core | Involvement of palliative care teams should begin early in the disease course to facilitate symptom management and build rapport over the course of the patient’s life | 90.7%  (49/54) |
| Core | After appropriate evaluation for treatable medical complications, management of pain should be a fundamental part of the care of patients with MPS III, with the aim of improving quality of life and maintaining mobility | 98.1%  (52/53) |
| Core | Recommend that clinicians are aware that pain in patients with cognitive impairment may be expressed via a variety of patient-specific pain behaviors and in non-classical ways | 98.3%  (57/58) |
| Core | There should be a low threshold for investigation of sources of pain in patients with escalating abnormal behaviors or acutely worsening sleep disturbances or signs of significantly increased agitation | 98.1%  (53/54) |
| Core | Investigation for sources of pain in children with escalating abnormal behaviors, acutely worsening sleep disturbances, or significantly increased agitation might include consideration of:   - Headaches (with consideration of elevated intracranial pressure) - Abdominal discomfort (ie acid reflux, ulcers, intestinal gas pain, constipation, etc) - Joint disease (ie arthralgia/arthritis, osteonecrosis of femoral head, etc) - ENT (ie otitis, sinusitis) - Dental-related pain | 98.0%  (50/51) |
| Core | In the case of persistent pain, distress or agitation for which outpatient evaluation has been unrevealing, admission to hospital for thorough, efficient medical workup is recommended, to include:   - Hip and spine X-ray - Dental examination (decay) - Abdominal imaging (constipation, other obstruction, etc) - Eye exam (consider elevated intraocular pressure) - Complete blood count with differential (infection, anemia) - Electrolytes (electrolyte imbalances, acidosis) - Brain scans if above are not revealing source of distress (hydrocephalus, intracranial bleed) | 86.0%  (43/50) |
| Core | Recommend that standardized pain assessments, appropriate for the patient’s cognitive level, and/or caregiver proxy assessments are included in the regular follow-up visits for patients with MPS III. For patients who have limited communicative ability, the NCCPC-R is recommended | 81.1%  (30/37) |

**N. Special education, physical, occupational, speech and complementary therapy interventions**

| **Core/ supplemental** | **Recommendation** | **Agree or strongly agree** |
| --- | --- | --- |
| Core | Therapeutic support interventions should strive for maintenance of skills rather than require improvement for continued service | 96.4%  (53/55) |
| Core | Rehabilitation therapeutic goals should focus on prolonging skills for as long as possible for improved quality of life and functional access to educational and social environments | 98.1%  (53/54) |
| Core | Rehabilitative therapies continue to be recommended even after decline in skills to maintain function at each interval for as long as possible | 97.2%  (51/55) |
| Core | Consultation and ongoing training on the use of augmentative and alternative communication (AAC) methods or devices which are fit to purpose for the individual patient is recommended. Note that children with MPS III may require longer and more intensive therapy to achieve successful use of these methods | 93.8%  (45/48) |
| Core | AAC methods should be initiated by a trained professional and then generalized to use in home and educational settings as quickly as possible | 93.6%  (44/47) |
| Core | Use of AAC methods and devices are recommended to be trialed and if feasible, instituted as early as possible during maximal cognitive capacity and even prior to loss of verbal speech | 89.4%  (42/47) |
| Core | Assessment of safe feeding abilities and skills should be performed if clinically indicated at diagnosis and monitored at least yearly and more frequently as disease symptoms progress | 98.0%  (48/49) |
| Core | Patients with deterioration in feeding skills and/or poor weight gain should be considered for referrals to a feeding therapist, nutritionist and to gastroenterology for consideration of nutritional supplementation and/or gastrostomy feeding device | 95.9%  (47/49) |
| Core | Diet/fluid modifications should be made using the International Dysphagia Diet Standardization Initiative (IDDSI) framework in consultation with a trained speech language feeding therapist | 97.3%  (36/37) |
| Core | Bedside clinical feeding and swallowing assessments should be done at mealtimes and in a variety of settings (home/school) to observe any behavioral/cognitive challenges around mealtimes | 97.8%  (44/45) |
| Core | If red flags are present for pharyngeal dysfunction (cough, wet voice, recurrent lower respiratory tract infections) recommend referral for Videofluoroscopic Swallowing Study (VFS) in consultation with a speech language feeding therapist | 95.1%  (39/41) |
| Core | Recommend that clinicians monitor for dysfluencies and speech apraxia which are noted symptoms of speech regression in MPS III patients | 95.6%  (43/45) |
| Core | Assess range of motion in upper and lower extremities at diagnosis and baseline visit to any new therapy provider and at regular intervals, at least every 6 months | 82.2%  (37/45) |
| Core | Recommend using consistent established measurement tools to track motor skills over time, choosing measures that are most appropriate for the child (eg Pediatric Evaluation of Disability Inventory [PEDI] or Functional Independence Measure [FIM]) | 81.0%  (34/42) |
| Core | Proactive support is recommended to obtain adaptive equipment such as wheelchair/medical stroller, stander, bath seat, activity chair, safety beds, lifts, specialized car seats, etc | 95.7%  (44/46) |
| Core | Orthotic bracing may be helpful for balance, foot/ankle positioning and to improve/maintain gait function and mobility longer | 86.7%  (39/45) |
| Core | Physiotherapy/physical therapy should be considered as early as possible and be performed regularly prior to the decline of gross motor skills to maintain mobility and function | 95.9%  (47/49) |

**O. General care**

| **Core/ supplemental** | **Recommendation** | **Agree or strongly agree** |
| --- | --- | --- |
| Core | Recommend multidisciplinary care model and medical home approach for long-term care coordination of patients with MPS III | 100%  (57/57) |
| Core | Growth should be monitored and plotted on MPS III-specific growth curves | 86.0%  (43/50) |
| Core | Immunizations should be given per routine standard of care schedule | 100%  (49/49) |
| Core | Yearly influenza vaccine is recommended | 97.9%  (47/48) |
| Core | Pneumovax 23 is recommended for patients with MPS III, in accordance with guidelines for those who are at increased risk of pneumococcal disease | 86.0%  (37/43) |
| Core | Management of hygiene and cessation of menstrual cycles may be considered via hormonal therapies if in concert with family’s care goals | 87.2%  (41/47) |
| Core | Supportive equipment needs should be discussed, and appropriate prescriptions/referrals made every 6 months (ie safe bathing equipment, safe vehicle transport chairs/ramps, seating in home, specialty beds, orthotics, etc) | 96.2%  (50/52) |
| Core | Environmental safety adaptations are recommended due to patients’ lack of safety sense, and cognitive decline with preserved motor skills | 100%  (57/57) |
| Core | Grouping procedures that require anesthesia is recommended as much as possible | 98.2%  (55/56) |
| Core | Cognitive impairment and the progressive nature of MPS III should not preclude a patients’ access to vision, hearing, behavioral or any other support services | 100%  (58/58) |
| Core | Recommend counseling caregivers on the natural history of disease progression in the absence of a disease-modifying treatment, at the time of diagnosis and periodically thereafter in order to plan appropriately and normalize their experiences with the disease process | 96.4%  (54/56) |
| Core | Proactive, intermittent assessment of caregivers’ anxiety, depression and chronic traumatic stress with appropriate referral is indicated | 100%  (57/57) |
| Core | Service needs of the family and child should be monitored regularly as these needs vary in intensity and type depending on the age and extent of the child’s disease progression | 98.3%  (57/58) |
| Core | Recommend connecting caregivers to relevant patient advocacy organizations, governmental support services and other community resources, checking back in to assess needs and additional referrals in an ongoing manner | 96.5%  (55/57) |
| Core | Recommend early quality educational experiences for maximal developmental gains and interval skill maintenance | 92.9%  (52/56) |
| Core | An individual aide in the school setting is recommended for maintaining safety of the child and others in the classroom as well as to maximize the child's attention and supports in educational tasks | 92.6%  (50/54) |

**P. Endocrinology**

| **Core/ supplemental** | **Recommendation** | **Agree or strongly agree** |
| --- | --- | --- |
| Core | Onset of puberty may be advanced in patients with MPS III. If signs of early puberty are present, referral to an endocrinology specialist is warranted. Use of GnRH agonist is not contraindicated in MPS III and should be considered in consultation with the family | 80.6%  (25/31) |
| Supplemental | Bone mineral density can be diminished in patients with advanced disease who are non-ambulatory. If there is concern for fracture risk, consider evaluation of bone mineral density (BMD). If abnormal, refer the patient to an endocrinology specialist. Use of bisphosphonates is not contraindicated in MPS III | 86.5%  (32/37) |

AAC, augmentative and alternative communication; ABA, applied behavioral analysis; ABR, auditory brainstem response; ADHD, attention deficit hyperactivity disorder; ALT, alanine aminotransferase; AST, aspartate aminotransferase; AV, atrioventricular; BMD, bone mineral density; BTE, behind the ear; CPAP, continuous positive airways pressure; DBS, dried blood spot; DMB, dimethylmethylene blue; ECG, electrocardiogram; ENT, ear, nose and throat; FIM, functional independence measure; GAG, glycosaminoglycan; GnRH, gonadotropin-releasing hormone; IDDSI, International Dysphagia Diet Standardization Initiative; IEP, individualized educational plan; IOP, intraocular pressure; MPS, mucopolysaccharidosis; MRI, magnetic resonance imaging; NCCPC-R, Non-communicating Children’s Pain Checklist – Revised; OME, otitis media with effusion; OSA, obstructive sleep apnea; PEPI, Pediatric Evaluation of Disability Inventory; PT, prothrombin time; PTT, partial thromboplastin time; VFS, Videofluoroscopic Swallowing Study
